# Mitophagy and Ubiquitination Coordinate Context‐Specific Mitochondrial Quality Control and EMT/MET Plasticity to Drive Cancer Cell Invasion

**DOI:** 10.1002/advs.202519792

**Published:** 2026-02-17

**Authors:** Bin‐Hsu Mao, Bo‐Kai Su, Ying‐Jan Wang, Ting‐Yuan Tu

**Affiliations:** ^1^ Department of Biomedical Engineering College of Engineering National Cheng Kung University Tainan City Taiwan; ^2^ Department of Biochemical Science and Technology National Taiwan University Taipei City Taiwan; ^3^ Department of Environmental and Occupational Health College of Medicine National Cheng Kung University Tainan City Taiwan; ^4^ Department of Medical Research China Medical University Hospital China Medical University Taichung City Taiwan; ^5^ Medical Device Innovation Center National Cheng Kung University Tainan City Taiwan

**Keywords:** EMT–MET plasticity, extracellular matrix mechanics, hypoxia and nutrient deprivation, mitophagy, mitochondrial dynamics, mitochondrial ROS, ubiquitination

## Abstract

Metastatic invasiveness emerges from coordinated intrinsic programs and microenvironmental cues that converge on mitochondrial quality control (MQC). Here, we use “context” to denote stage‐ and site‐aware constellations of tumor‐intrinsic states (e.g., mtROS tone, mtDNA integrity, epigenetic wiring, cellular stiffness, oncogenic mutations) and extrinsic landscapes (oxygen–nutrient availability, ECM mechanics, stromal/inflammatory signals). These axes jointly shape mitochondrial adaptation by tuning bioenergetics, redox balance, metabolic plasticity, fission–fusion dynamics, mechanosensitive hubs, and Ca^2^^+^ homeostasis. As pressures intensify, mitochondrial vulnerabilities—such as mtDNA compromise and mtUPR activation—signal the engagement of mitophagy to preserve organelle fitness under stress. Through these coupled changes in mitochondrial performance and stress responses, context governs EMT/MET plasticity and transitions across migratory, invasive, and proliferative states. Mechanistically, ubiquitin conjugation, via E3 ligases and deubiquitinases, serves as an integrating conduit that links mitochondrial remodeling and mitophagy to cytoskeletal reprogramming and invasive behavior. This ubiquitin–mitochondria interface therefore represents a coherent therapeutic entry point; translational strategies including PROTAC‐enabled targeting and selective E3/DUB or mitophagy‐pathway modulators may rebalance pathological ubiquitin signaling, restore mitochondrial homeostasis, and constrain tumor dissemination.

## Graphical abstract

Metastatic invasion emerges when tumor‐intrinsic programs and microenvironmental forces converge on mitochondrial quality control. A context‐stratified framework links mtROS, mtDNA integrity, mechanics and nutrient/oxygen cues to fission–fusion remodeling, mtUPR and mitophagy. Ubiquitin “writers” and “erasers” translate these pressures into EMT/MET switching, revealing actionable intervention points such as E3/DUB modulators and PROTAC‐like tools.

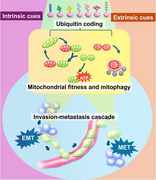

**Abbreviations**
ABALON: apoptotic BCL2L1‐antisense long non‐coding RNA
AMPK: AMP‐activated protein kinase
AP‐1: activator protein 1
ARIH1: ariadne RBR E3 ubiquitin protein ligase 1
ATF: activating transcription factor
ATOX1: antioxidant 1 copper chaperone
ATP7A: ATPase copper‐transporting alpha
ATP7B: ATPase copper‐transporting beta
BAP1: BRCA1‐associated protein 1
BBA: Biochimica et Biophysica Acta
BNIP3: BCL2/adenovirus E1B 19 kDa‐interacting protein 3
BORIS: Brother of the regulator of imprinted sites
β‐TrCP: β‐transducin repeat‐containing protein
CAFs: cancer‐associated fibroblasts
CAMs: cancer‐associated macrophages
CDK1: cyclin‐dependent kinase 1
CDK1/5: cyclin‐dependent kinases 1/5
CHAC2: ChaC glutathione‐specific gamma‐glutamylcyclotransferase 2
CHIP: C‐terminus of Hsc70‐interacting protein
CHOP: C/EBP homologous protein
circRNAs: circular RNAs
CLEAR: coordinated lysosomal expression and regulation
ClpP: caseinolytic mitochondrial matrix peptidase proteolytic subunit
CMS1: consensus molecular subtype 1
COX17: cytochrome c oxidase copper chaperone
CSC: cancer stem cell
CTCF: CCCTC‐binding factor
DCP2: mRNA‐decapping enzyme 2
DNMTs: DNA methyltransferases
Drp1: dynamin‐related protein 1
DUBs: deubiquitinases
ECM: extracellular matrix
EMT: epithelial‐to‐mesenchymal transition
EMT‐TFs: EMT transcription factors
ER: endoplasmic reticulum
ERK1/2: extracellular signal‐regulated kinases 1/2
ETC: electron transport chain
ETS1: ETS proto‐oncogene 1, transcription factor
EVs: extracellular vesicles
FAK: focal adhesion kinase
FAO: fatty acid oxidation
FBXL14: F‐box and leucine‐rich repeat protein 14
FBXL2: F‐box and leucine‐rich repeat protein 2
FBXO11: F‐box only protein
FOXO3: forkhead box O3
FUNDC1: FUN14 domain‐containing protein 1
GABARAP: LC3‐or gamma‐aminobutyric acid receptor‐associated protein
Gp78: glycoprotein 78
GPX4: glutathione peroxidase 4
HCC: hepatocellular carcinoma
HDAC: histone deacetylase
HSP: heat shock protein
Hsp70: heat shock protein family A
HSPA8: heat shock protein family A member 8
IGF2BP3: insulin‐like growth factor 2 mRNA‐binding protein 3
IL: interleukin
IP3R3: inositol 1,4,5‐trisphosphate receptor type 3
ITGA3: integrin subunit alpha 3
JNK: c‐Jun N‐terminal kinase
KEAP1: Kelch‐like ECH‐associated protein 1
LINC: linker of nucleoskeleton and cytoskeleton
LIR: LC3‐interacting region
lncRNA: long non‐coding RNA
LONP1: lon peptidase 1, mitochondrial
LOX: lysyl oxidase
m6A: N6‐methyladenosine
MAMs: mitochondria‐associated membranes
MARCH5: membrane‐associated RING‐CH protein 5
MCU: mitochondrial calcium uniporter
MDM2: mouse double minute 2 homolog
MDV: mitochondria‐derived vesicle
MET: mesenchymal‐to‐epithelial transition
MET‐2: methyltransferase‐2
METTL3: methyltransferase‐like 3
Mff: Fis1/mitochondrial fission factor
MFN1/2: mitofusin 1/2
MICOS: mitochondrial contact site and cristae organizing system
MID49/51: mitochondrial dynamics proteins of 49/51 kDa
MIF: macrophage migration inhibitory factor
miRNAs: microRNAs
Miro1: mitochondrial Rho GTPase 1
mitoKATP: mitochondrial ATP‐sensitive potassium channel
MMP: matrix metalloproteinase
MQC: mitochondrial quality control
MSK1: mitogen‐ and stress‐activated protein kinase 1
MST3: mammalian STE20‐like protein kinase 3
mtDNA: mitochondrial DNA
MTG1: mitochondrial GTPase 1
mTOR: mechanistic target of rapamycin
mTORC1: mTOR complex 1
mtROS: mitochondrial reactive oxygen species
mtUPR: mitochondrial unfolded protein response
MUL1: mitochondrial ubiquitin ligase 1
MYSM1: Myb‐like, SWIRM, and MPN domains 1
NBR1: next to BRCA1 gene 1 protein
NCLX: mitochondrial Na^+^/Ca^2^^+^/Li^+^ exchanger
ncRNAs: non‐coding RNAs
NDP52: nuclear dot protein 52 kDa
NF‐κB: nuclear factor‐kappa B
NLRP3: NOD‐like receptor family, pyrin domain containing 3
Nrf2: nuclear factor erythroid 2–related factor 2
OMM: outer mitochondrial membrane
OPA1: optic atrophy 1
OPTN: optineurin
OTUD6A: OTU deubiquitinase 6A
OVOL2: OVO‐like zinc finger 2
OXPHOS: oxidative phosphorylation
PARK2: Parkin RBR E3 ubiquitin protein ligase
PARL: presenilin‐associated rhomboid‐like protein
PD‐L1: programmed death‐ligand 1
PDEs: patient‐derived explants
PDH: pyruvate dehydrogenase
PDO: patient‐derived organoid
PGAM5: phosphoglycerate mutase family member 5
PHB2: prohibitin 2
PHD: prolyl‐hydroxylase domain
PIEZO1: Piezo‐type mechanosensitive ion channel component 1
PIKFYVE: phosphoinositide kinase, FYVE‐type zinc finger containing
PINCH1: particularly interesting new cysteine–histidine rich protein 1
PKA: protein kinase A
PK/PD: pharmacokinetics/pharmacodynamics
PRC1: polycomb repressive complex 1
PROTAC: proteolysis‐targeting chimera
PTEN: phosphatase and tensin homolog
RAC1: Ras‐related C3 botulinum toxin substrate 1
RNF8: ring finger protein 8
ROS: reactive oxygen species
RREB1: Ras‐responsive element‐binding protein 1
SCF: SKP1–CUL1–F‐box
SFXN1: sideroflexin‐1
SIRT3: sirtuin 3
Skp2: S‐phase kinase‐associated protein 2
SLC7A11: solute carrier family 7 member
Smurf1: SMAD ubiquitination regulatory factor 1
SNAI1: Snail family transcriptional repressor 1
SOD2: superoxide dismutase 2
ST2: suppression of tumorigenicity 2
TBK1: TANK‐binding kinase 1
TCF4: transcription factor 7‐like 2
TEAD: TEA domain transcription factor
TF: transcription factor
TFCP2: transcription factor CP2
TFEB: transcription factor EB
TGF‐β: transforming growth factor‐beta
TIGAR: TP53‐induced glycolysis and apoptosis regulator
TMEs: tumor microenvironments
TRAF6: TNF receptor–associated factor 6
TRIM: tripartite motif
TWIST1: Twist‐related protein 1
Ub: ubiquitin
UBA: ubiquitin‐associated domain
UBAN: ubiquitin‐binding in ABIN and NEMO
UBL: ubiquitin‐like
UBL‐5: ubiquitin‐like protein 5
UCHL1: ubiquitin C‐terminal hydrolase L1
ULK1: unc‐51‐like kinase 1
UPS: ubiquitin–proteasome system
USF2: upstream stimulatory factor 2
USP: ubiquitin‐specific protease
5′‐UTR: 5′ untranslated region
VDAC1: voltage‐dependent anion channel 1
WTAP: Wilms tumor 1–associating protein
YAP: Yes‐associated protein
ZEB1: zinc finger E‐box binding homeobox 1
ΔΨm: mitochondrial membrane potential

## 1 Introduction

Metastasis—the spread of cancer cells beyond the primary tumor to remote organs—is a complex, multi‐step process and the leading cause of cancer mortality, accounting for over 90% of cancer‐associated deaths [1]. Mechanistically, the cascade centers on epithelial–mesenchymal plasticity, as many epithelial‐lineage malignancies navigate an epithelial‐to‐mesenchymal transition (EMT)–mesenchymal‐to‐epithelial transition (MET) continuum—loosening epithelial constraints to invade and disseminate, and frequently restoring epithelial attributes via MET at distant sites—such that this bidirectional cell‐state flexibility, as opposed to a unidirectional EMT, acts as a principal driver of colonization [2]. Within tumor microenvironments (TMEs) marked by low oxygen, inflammatory mediators, and mechanically stiffened matrices, stabilized hypoxia‐inducible factor‐1 alpha (HIF‐1α), together with activated nuclear factor‐kappa B (NF‐κB) and transforming growth factor‐beta (TGF‐β) signaling, orchestrates cytoskeletal remodeling that facilitates invasion [3]. Beyond the primary lesion, successful dissemination is governed by “seed–soil” compatibility, pre‐metastatic niche priming, and organ‐specific tropism, which jointly determine whether scarce tumor cells in circulation survive, arrest within distant capillary beds, and form secondary lesions [4]. Within this interconnected biological system, mitochondria operate as more than ATP factories, fulfilling roles as strategically localized signaling hubs that reprogram bioenergetics, set oxidative setpoints, and integrate Ca^2^^+^ flux into membrane–cytoskeletal networks—features enriched at advancing edges, maintain stress‐resilient motility, and promote growth of early metastatic colonies [5]. However, despite extensive characterization of EMT/MET transitions and TME‐related signaling networks, how cancer cells translate these cues via mitochondrial quality‐control (MQC)—particularly its ubiquitin (Ub)‐directed arm—remains largely unexplored. Recognizing this mechanistic blind spot recasts mitochondria as context‐responsive organelles whose maintenance machinery couples bioenergetic resilience to the phenotypic switches that drive invasion and metastasis [6].

A growing body of evidence indicates that metastatic fitness depends on “context‐dependent” mitochondrial control, whereby tumor cells dynamically adjust mitochondrial metabolism, spatial deployment, and functional maintenance pathways in response to both cell‐intrinsic programs and microenvironmental constraints. Against this backdrop of contextual regulation, metastatic cells adaptively rewire mitochondria along diverse mechanistic axes: they alternate between oxidative phosphorylation (OXPHOS) and glycolysis, reposition mitochondria toward actin‐rich protrusions, and generate spatially confined mitochondrial reactive oxygen species (mtROS) microdomains that reinforce HIF‐1α/NF‐κB signaling. In a coordinated way, mitochondrial compartments govern Ca^2^^+^ flux to support actin‐cytoskeleton remodeling, in turn driving invadopodia assembly and the pericellular matrix turnover that drives invasive efficiency and metastatic potential [7]. Selective mitochondrial clearance via mitophagy is central to this adaptive resilience—buffering oxidative stress and preserving OXPHOS capacity—yet its contribution to metastatic behavior varies over the pathological trajectory of tumor evolution [8]. Consistent with this stress‐tuned rationale, evidence across heterogeneous experimental systems—including 2D cell‐line monolayers and 3D spheroid/organoid platforms, ex vivo tissue preparations, and validated in vivo metastasis assays—demonstrates that mitophagy exerts stage‐dependent effects: it can restrain early migratory initiation yet support later metastatic outgrowth by safeguarding mitochondrial fitness under escalating metabolic and mechanical demands [6, 9, 10, 11, 12]. Importantly, these adaptations are not merely mitophagy‐driven but are also shaped by ubiquitination, a central regulatory axis within MQC that controls mitochondrial tagging, turnover, and repair [13]. These cross‐model insights uncover previously overlooked regulatory intersections where tumor‐intrinsic regulatory circuits—such as mitochondrial DNA (mtDNA)‐upkeep pathways (copy‐number control and fidelity), epigenetic and epitranscriptomic regulation, and the machinery orchestrating mitochondrial network architecture—align with microenvironment‐driven pressures originating from limited oxygen availability, nutrient deprivation, matrix‐driven mechanical stiffness, hemodynamic or interstitial shear stress, and stromal‐derived inflammatory or metabolic cues [14, 15, 16].

**FIGURE 1:**
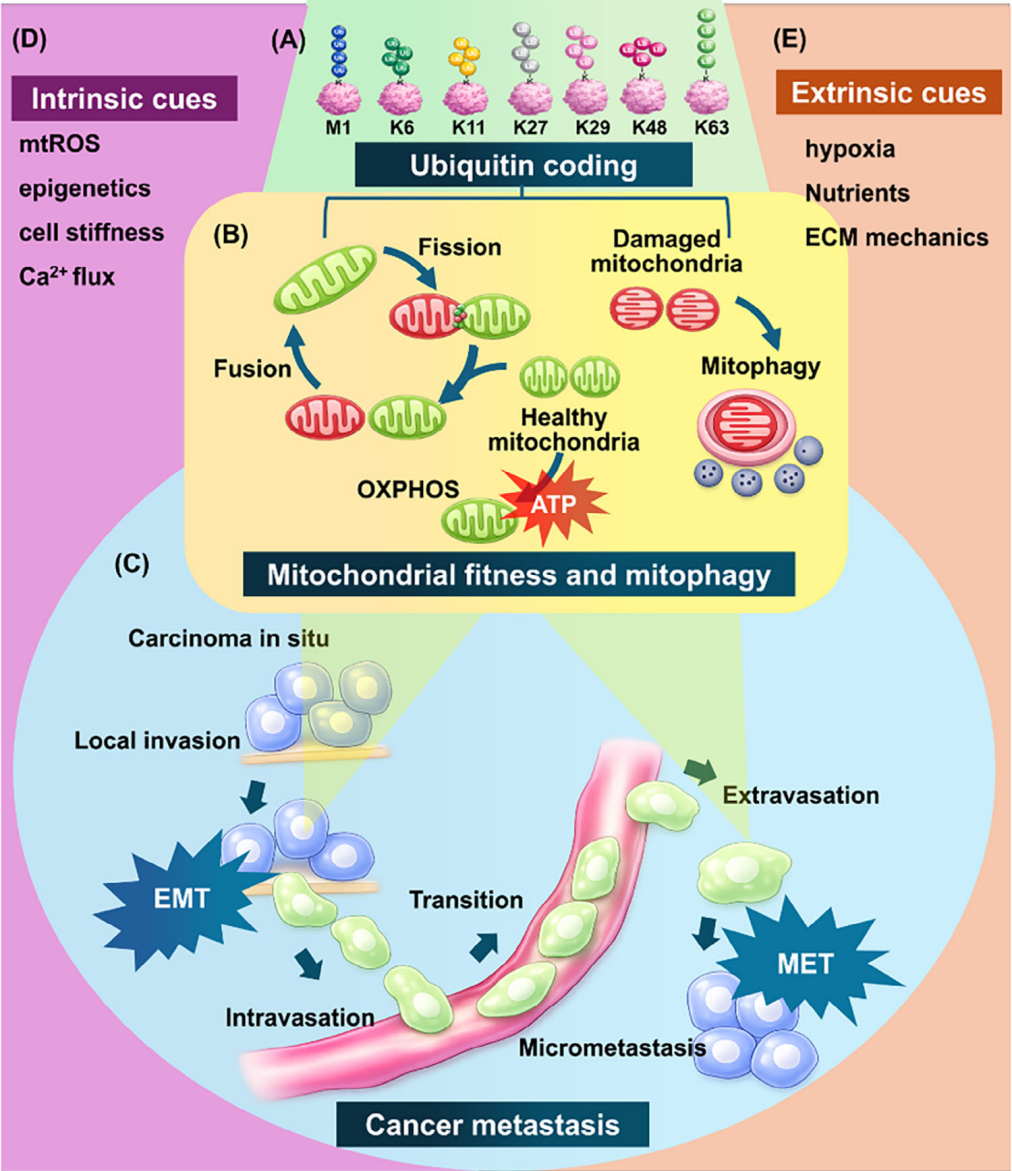
A context‐integrated framework linking ubiquitin coding to mitochondrial quality control and EMT/MET plasticity during cancer metastasis. (A) Ubiquitin coding logic: distinct Ub chain/linkage types (M1, K6, K11, K27, K29, K48, and K63) encode degradative and non‐degradative signals that route mitochondrial and signaling substrates toward proteostasis, remodeling, or selective clearance. (B) Mitochondrial fitness and mitophagy: fusion–fission dynamics and oxidative phosphorylation (OXPHOS) sustain ATP output in healthy mitochondria, whereas damaged mitochondria are selectively eliminated via mitophagy to preserve organelle quality under stress. (C) Metastatic cascade model depicting the interface between mitochondrial fitness maintenance and epithelial–mesenchymal plasticity across sequential stages, including carcinoma in situ, EMT‐linked local invasion, intravasation, transit, extravasation, and micrometastasis with MET. (D) Intrinsic cues (e.g., mtROS, epigenetic state, cell stiffness, Ca^2^^+^ flux) and (E) extrinsic cues (e.g., hypoxia, nutrient availability, ECM mechanics) converge on Ub‐coded mitochondrial regulation to shape context‐dependent EMT/MET switching and dissemination outcomes.

Before further developing this concept, it is useful to clarify what “context” encompasses in the present framework. As depicted in Figure 1, the convergence of multiple contextual dimensions on MQC pathways motivates the framework described below. To formalize this perspective, we advance the notion of “context” as a tripartite framework comprising (i) cell‐autonomous regulatory factors [7] and (ii) microenvironment‐imposed cues [8], with (iii) disease stage‐ or locale‐associated features serving as secondary modifiers [17, 18]. Recent studies support this framework by documenting that MQC programs—spanning stress‐responsive regulation of mitochondrial morphodynamics, subcellular positioning, and organ‐specific metabolic rewiring—undergo iterative reconfiguration as extracellular‐matrix (ECM) anchorage is lost, invasive transit occurs, and colonizing growth begins [19, 20, 21, 22]. These regulatory mechanisms likewise diverge across metastatic sites, underscoring adjustment in response to the situational pressures and microenvironmental constraints tied to different phases and anatomical locations of metastasis. These contextual axes shape the metabolic and mechano‐inflammatory conditions that mitochondria adopt, thereby influencing microdomain‐level ATP, ROS, and Ca^2^^+^ signaling profiles that drive cytoskeletal reorganization [23, 24].

Crucially, ubiquitination serves as an information‐processing layer that translates these context axes into executable MQC actions. Cell‐intrinsic programs and microenvironmental constraints can reprogram the recruitment, activity, and sub‐mitochondrial access of ubiquitin “writers” (E3 ligases) and “erasers” (deubiquitinases (DUBs)), while simultaneously reshaping which outer‐membrane substrates are exposed, clustered, or shielded. Through these changes, mitochondrial ubiquitin architectures—including linkage composition and signal persistence—are remodeled and then interpreted by ubiquitin‐binding adaptors to coordinate two coupled outputs: remodeling and redeployment of functional organelles, and selective elimination of compromised segments through mitophagy [25]. In PINK1/Parkin settings, phosphorylation‐driven feed‐forward ubiquitin tagging can rapidly amplify mitochondrial labeling, whereas chain topology and counteracting DUBs help tune cargo selectivity and clearance efficiency [26]. Downstream of this ubiquitin‐directed triage, mitochondrial bioenergetic and signaling capacity becomes spatiotemporally available to the actin remodeling machinery, which directs the formation of lamellipodia, filopodia, invadopodia, or blebs and modulates mesenchymal–amoeboid migratory plasticity [27]. By this route, stage‐ and site‐specific shaping of mitochondrial activity patterns underlies the multifaceted invasion–metastatic phenotypes exhibited across tumor niches [28, 29, 30].

Recent reviews have comprehensively surveyed MQC is a broad homeostatic network, spanning selective autophagy programs and organelle surveillance logic across physiology and disease [31], as well as mitochondria‐derived vesicle (MDV) trafficking as an additional, mitophagy‐adjacent route for mitochondrial cargo triage and inter‐organelle signaling [32]. In contrast, the present review focuses specifically on the ubiquitin–mitochondria interface as a metastasis‐relevant control layer: we emphasize how ubiquitin “writing/erasing” architectures on the mitochondrial surface couple to mitophagy flux to tune mitochondrial stress outputs (redox, bioenergetic buffering, proteostasis signaling) and accordingly gate EMT/MET switching and metastatic state transitions. By organizing metabolic and biophysical pressures as upstream inputs and ubiquitin‐dependent MQC as an executable decision module, this framework aims to clarify when and why mitochondrial clearance circuitry is co‐opted to support dissemination, therapy tolerance, and colonization rather than simply serving as damage removal.

To organize these concepts within a cohesive conceptual scheme, the remainder of this review is organized as follows. Section 2 evaluates how invasion‐demanded mitochondrial adaptations—encompassing metabolic rewiring, ROS/Ca^2^^+^ microdomain generation, spatial redistribution, and mechano–ionic coupling—engender dependence on stress‐ready MQC systems. Section 3 then analyzes how mitophagy fulfills these invasion‐imposed MQC demands, emphasizing its context‐contingent engagement across EMT/MET transitions, loss of ECM anchorage, invasive migration, and early metastatic outgrowth. Section 4 advances this foundation by detailing how ubiquitination constitutes a central coordinating layer that governs mitochondrial labeling, clearance pathways, proteostatic balance, mtUPR engagement, and organelle architectural remodeling, linking cell‐intrinsic molecular settings to extrinsic pressures. To translate these mechanistic insights into a scenario of therapeutic intervention, Section 5 highlights anti‐cancer prospects at the ubiquitin–mitophagy/mitochondria crossroads, covering current and evolving modalities that therapeutically modulate key MQC nodes—including E3 ligases, DUBs, and organelle‐resident QC effectors—and outlining outstanding challenges and future directions for metastasis‐oriented therapy. In aggregate, these sections offer an integrated, multi‐tiered perspective on how stage‐ and site‐specific mitochondrial fitness maintenance synergizes with EMT/MET remodeling to sustain cancer cell invasion and metastatic competence.

## 2 Context‐Dependent Mitochondrial Regulation of EMT and Metastatic Behavior in Cancer

Mitochondria are no longer best viewed as static bioenergetic engines; instead, they function as adaptable hubs whose structure, positioning, and output patterns can dictate whether tumor cells acquire and sustain migratory and invasive competence. In addition to meeting energetic demand, mitochondria generate signaling‐competent ROS that set signaling amplitude [33] and coordinate intracellular Ca^2^^+^ homeostasis through regulated uptake and efflux [34], while also orchestrating stress‐response programs that preserve organelle fitness under pressure [35]. In accordance with the framework described in the Introduction, “context” represents the integrated effects of tumor‐intrinsic state, TME‐derived inputs, and stage‐/site‐specific constraints. Collectively, these factors determine how mitochondrial ATP availability, local redox microdomains, and Ca^2^^+^ trafficking are decoded—either as EMT‐permissive cues that support dissemination or as limiting signals that favor MET‐compatible outgrowth.

Mechanistically, Section 2 organizes mitochondrial control of metastasis into four interconnected dimensions: (i) energetic and metabolic flexibility that establishes the ATP budget for cytoskeletal remodeling and matrix invasion, (ii) graded and spatially confined mtROS signaling that repatterns EMT and adhesion programs, (iii) network reorganization and polarized redistribution that deliver ATP/ROS to protrusive zones, and (iv) mechano–ionic coupling in which Ca^2^^+^ flux integrates with ECM forces to fine‐tune contractility and migration efficiency [23, 36, 37, 38]. Additional layers—mtDNA copy‐number/heterogeneity, mtUPR enactment, and intercellular mitochondrial transfer—further recalibrate respiratory capacity and stress tolerance across metastatic stages [39]. Subsections 2.1–2.5 then consider these axes in sequence, and Table 1 outlines mitochondrial features most reliably linked to the invasion–metastasis cascade. For an at‐a‐glance summary of the directional effects of major mitochondrial signaling modes (ROS, Ca^2^^+^, mtDNA, mtUPR) and the ubiquitin system on EMT/MET plasticity, see Table 8.

**TABLE 1:** Contextual determinants of mitochondrial impact on tumor dissemination and colonization.

Context	Effect on metastasis	Strength of evidence	Reference
Mitochondrial bioenergetics (OXPHOS activity)	OXPHOS enhances persistent migration and colonization; glycolysis may dominate at early stages.	Well‐supported	[41, 72]
Mitochondria‐driven metabolic plasticity	Metabolic plasticity allows cancer cells to switch between glycolysis and OXPHOS during metastatic progression.	Emerging evidence	[40]
Mitochondrial ROS levels	Moderate levels promote metastasis via EMT and signaling activation; excessive levels inhibit metastasis via oxidative damage.	Well‐supported	[69, 70]
Mitochondrial fission vs. fusion dynamics	Increased fission promotes cellular plasticity and migration; fusion maintains epithelial traits.	Well‐supported	[71, 72]
Mitochondrial trafficking and positioning	Trafficking mitochondria to the leading edge supports local ATP delivery and invasion.	Well‐supported	[71, 72]
Interaction with mechanical forces (e.g., ECM stiffness)	Stiffened ECM modulates mitochondrial positioning and dynamics to influence migratory behavior.	Emerging evidence	[80, 81, 82]
Mitochondrial Ca^2^^+^ uptake	Mitochondrial Ca^2^^+^ uptake regulates bioenergetics, ROS, and signaling critical for migration and invasion.	Well‐supported	[96, 97, 98]
Mitochondrial DNA content and integrity	Elevated mtDNA content promotes metabolic fitness and metastasis; specific mtDNA mutations can suppress metastasis.	Well‐supported	[41, 64]
Mitochondrial unfolded protein response (mtUPR)	mtUPR enhances mitochondrial repair and stress resistance, potentially promoting metastatic fitness.	Emerging evidence	[35, 115]

### 2.1 The Role of Mitochondrial Energetics and Metabolic Plasticity in Tumor Spread

To push through metastatic bottlenecks along the cascade, cancer cells have to maintain robust bioenergetic output and metabolic flexibility. The Warburg effect—glycolytic ATP synthesis even under oxygen‐replete conditions—reflects only one aspect of tumor metabolism. Many malignant cells retain functional mitochondria and dynamically toggle between OXPHOS and glycolysis in response to TME‐derived inputs such as oxygen tension, nutrient access, and mechanical stress, thereafter meeting the increased ATP requirements of actomyosin contractility, ECM degradation, and invasive motility [40]. This perspective repositions invasion–dissemination as a phase where mitochondrial fitness is often preserved—and can be amplified—rather than uniformly suppressed, supporting the energetic burden of escape and colonization [23]. Building on this metabolic‐plasticity framework, elevated mtDNA copy number—which can augment OXPHOS flux—has been associated with more pronounced growth and metastatic outgrowth in respiration‐reliant cancers [41]. More broadly, these observations argue against a fixed metabolic identity during metastasis, suggesting instead that disseminating cells occupy a continuum between glycolysis and OXPHOS and retune ATP‐generating circuitry to match prevailing constraints [16]. Recent studies across tumor types extend this continuum model by showing that metastatic capacity can be upheld by selectively reinforcing—and context‐tuning—respiratory function. On a mechanistic, basis, this can entail coupling fatty acid oxidation (FAO) to OXPHOS with nuclear factor erythroid 2–related factor 2 (Nrf2)–coordinated cytoprotective buffering, reliance on complex I–supported respiration in oxygen‐rich niches, and mitochondrial RNA–dependent adjustments that optimize respiration to secure ATP under fluctuating pressures [42, 43, 44]. Collectively, these routes underscore a dual implementation of metabolic flexibility: cells can re‐balance glycolysis and OXPHOS at the single‐cell level, and invading cohorts can distribute energetic tasks across specialized sub‐states to sustain protrusive activity and matrix remodeling.

How this dual‐mode plasticity is implemented becomes most evident during collective invasion. At this scale of multicellular movement, invasive assemblies distribute energetic workloads across subpopulations: “leader” cells preferentially leverage pyruvate dehydrogenase (PDH)–supported mitochondrial respiration to fuel front‐oriented protrusion, while “follower” cells shift toward a more glycolytic program—jointly creating a metabolic labor partitioning that sustains cohesive invasion [45]. Layered on top of this intracoalition energy sharing, metastatic progression imposes its own environmental “weighting” that reshapes which pathway carries the load. At the primary site and during dissemination—conditions dominated by low oxygen and mechanical strain—cells often emphasize rapid fermentative ATP production to satisfy short‐horizon needs; after arrival in well‐oxygenated secondary niches, the balance can shift back toward oxidative metabolism to sustain durable expansion [46]. This stage‐tuned signature is further influenced by non‐hypoxic cues—such as matrix stiffness, nutrient availability, and cellular redox tone—which jointly bias how strongly cells engage mitochondrial energy pathways and, in turn, determine metastatic fitness [47]. Importantly, cell‐intrinsic perturbations can also drive this reweighting: in select high‐stress settings, mitochondrial OXPHOS dysfunction can paradoxically facilitate treatment persistence [48]. In parallel, oncogenic programs and mechanically imposed energetic load (e.g., stiffness‐ or confinement‐derived demand) act as convergent dials that redistribute ATP production between respiratory and fermentative routes, favoring EMT‐permissive bioenergetic configurations that underwrite cell movement, tissue invasion, and stress endurance [49, 50, 51]. Overall, metastatic progression selects for flexible energy allocation that pairs rapid force‐support with mitochondrial endurance, and this bioenergetic flexibility inherently specifies the amplitude and localization of mitochondrial redox cues. Subsection 2.2 next examines how graded mtROS signaling converts these metabolic states into invasion‐promoting transcriptional and cytoskeletal outputs.

### 2.2 Graded mtROS and Their Roles in Tumor Dissemination

mtROS function as a second messenger that connects mitochondrial metabolism to pro‐metastatic signaling, including pathways governed by HIF‐1α, NF‐κB, and Src‐family kinases [52]. Within a physiological range, intermediate mtROS elevations can activate EMT‐linked programs, promote ECM remodeling, and enable cytoskeletal plasticity required for invasive movement through the pericellular environment [23]. Recent studies indicate that moderate, signaling‐range mtROS function less as metabolic byproducts and more as tunable redox inputs that are funneled through a limited set of relay modules to promote invasive programs. Mechanistically, these relays include (i) post‐translational phospho‐switch networks that couple mtROS to mechanically gated transcriptional responses—to give one example, a presenilin‐associated rhomboid‐like protein (PARL)–phosphoglycerate mutase family member 5 (PGAM5)–mammalian STE20‐like protein kinase 3 (MST3) module that converges on Yes‐associated protein (YAP) activation state [53], (ii) oxidant‐sensing dependent transcriptional responses that amplify motility‐linked gene expression, with NF‐κB and activator protein 1 (AP‐1) as representative nodes [54], and (iii) set‐point controllers that keep reactive oxygen species (ROS) within a permissive window to stabilize EMT‐leaning signaling, for example via TP53‐induced glycolysis and apoptosis regulator (TIGAR)‐mediated calibration [55]. Together, this integrated evidence frames mtROS as an adjustable rheostat whose amplitude and intracellular routing determine whether redox signals reinforce EMT‐associated migration and invasion.

The overall effect of mtROS is context‐dependent since baseline redox tolerance is conditioned by intrinsic features—electron transport chain (ETC) coupling, metabolic adaptability, and redox‐protective buffering driven by Nrf2, superoxide dismutase 2 (SOD2), and glutathione peroxidase 4 (GPX4)—while extrinsic cues determine when mtROS is interpreted as pro‐invasive signaling [56]. Three mechanistic units illustrate this gating: (i) oxygen fluctuation (hypoxia/reoxygenation) can elicit complex III–derived ROS bursts that activate invasion programs [57]; (ii) mechanical stiffening can stimulate integrin–focal adhesion kinase (FAK)–mtROS feedback circuitry to foster invadopodia assembly [58]; and (iii) stromal‐derived cytokines, including fibroblast‐lineage TGF‐β and interleukin (IL) family signals (e.g., IL‐6), can recalibrate redox poise to stabilize EMT and support tumor outgrowth [59]. Mechanistically, modest mtROS can stabilize even under normoxia to induce Twist‐related protein 1 (TWIST1), Snail family transcriptional repressor 1 (SNAI1), and zinc finger E‐box binding homeobox 1 (ZEB1)—core EMT transcription factors (EMT‐TFs)—while in parallel activating Src family kinases and Ras‐related C3 botulinum toxin substrate 1 (RAC1) to remodel actin and promote extracellular matrix degradation [60, 61].

Critically, mtROS exhibits a biphasic association with metastasis: driving mtROS beyond a stress breakpoint—or unduly suppressing it below a pro‐migratory window—can restrain disseminative competence. Excess ROS damages macromolecules, compromises mitochondrial function, and can engage apoptosis via cytochrome c release and caspase activation, in turn limiting metastatic establishment [62]. In vivo, mitochondria‐targeted antioxidant strategies can suppress recurrence and colonization by reducing pro‐metastatic mtROS signals (e.g., MitoQ in breast‐cancer models) [63]. Conversely, severe ETC dysfunction can generate ROS overload and cytotoxicity that blocks metastatic outgrowth (e.g., pathogenic mtDNA mutations limiting melanoma lung metastasis) [64].

**FIGURE 2:**
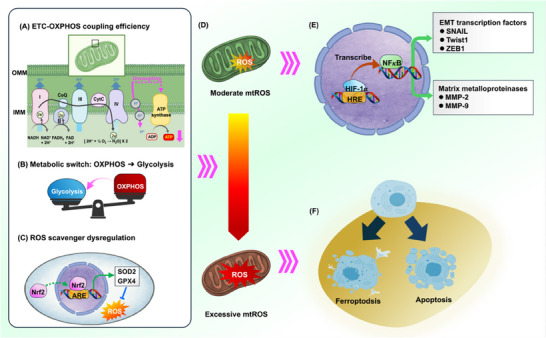
Biphasic control of metastatic signaling and cell fate by mitochondrial ROS. (A) ETC–OXPHOS coupling regulates basal ROS production by minimizing electron leakage during oxidative phosphorylation. (B) Under metabolic stress, tumor cells shift from OXPHOS to glycolysis, contributing to redox imbalance. (C) Impaired Nrf2‐mediated antioxidant signaling (e.g., SOD2 and GPX4) further elevates mtROS levels. (D) Moderate mtROS serve as signaling intermediates, enhancing the transcriptional activity of HIF‐1α and NF‐κB. (E) Activation of HIF‐1α and NF‐κB induces EMT‐TFs (e.g., SNAI1, TWIST1, and ZEB1) and MMPs (e.g., MMP‐2 and MMP‐9), facilitating invasion. (F) Excessive mtROS trigger mitochondrial dysfunction and activate cell death pathways—namely apoptosis and ferroptosis—thus serving as a redox‐imposed barrier to metastasis.

Moreover, ROS overload can potentiate ferroptosis, offering an anti‐metastatic route that may selectively eliminate highly invasive, apoptosis‐resistant cells [65], with multiple axes converging on GPX4, solute carrier family 7 member 11 (SLC7A11), and iron‐handling pathways [66, 67, 68]. Overall, mtROS act as a thresholded cue: localized, mid‐range signals bias cells toward EMT‐associated invasion, whereas extreme oxidant burden triggers apoptosis/ferroptosis and curtails outgrowth [69, 70]. Figure 2 integrates the upstream determinants that tune mtROS output—ETC performance, metabolic rerouting, and antioxidant capacity—into a unified redox‐gating model. Building on this, Subsection 2.3 examines how spatial positioning of mitochondria establishes the subcellular microdomains that execute these outputs during migration.

### 2.3 Mitochondrial Network Remodeling and Polarized Positioning During Cancer Cell Metastasis

**FIGURE 3:**
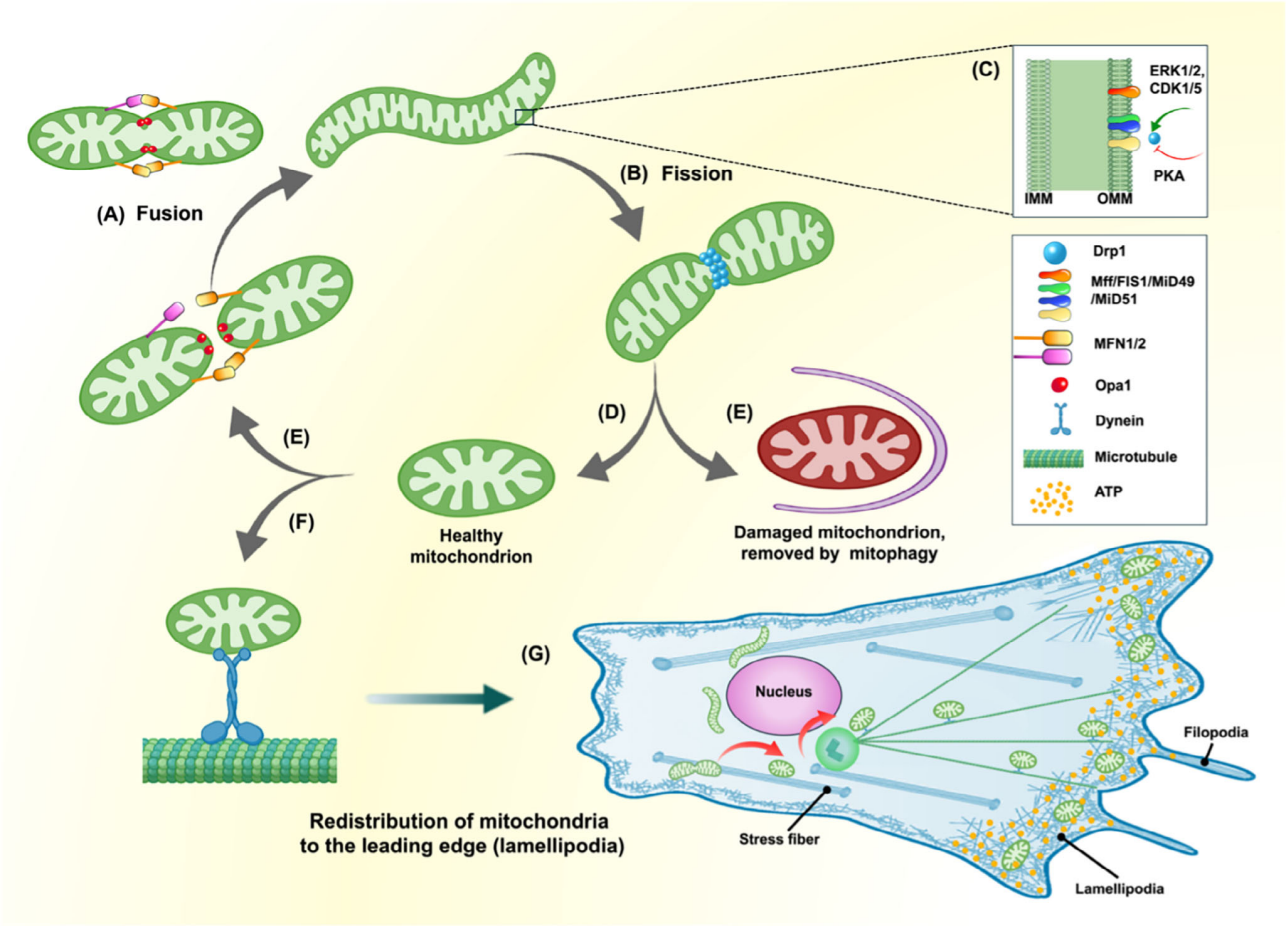
Coordination of mitochondrial dynamics, quality control, and polarized redistribution during cancer cell invasion. (A) Mitochondrial fusion is mediated by MFN1/2 (outer mitochondrial membrane; OMM) and OPA1 (inner mitochondrial membrane; IMM), restoring mitochondrial integrity and forming elongated, interconnected networks. (B) Mitochondrial fission is initiated by the recruitment of Drp1 to the outer mitochondrial membrane (OMM) through adaptors including Fis1, Mff, and mitochondrial dynamics proteins of 49/51 kDa (MID49/51), resulting in organelle fragmentation. (C) Drp1 activity is regulated via phosphorylation by upstream kinases such as ERK1/2 and CDK1/5; PKA‐mediated phosphorylation counteracts this effect. (D) Fission yields two daughter mitochondria, which are differentially fated based on their functionality and membrane potential. (E) Damaged mitochondria with depolarized membrane potential are selectively removed via mitophagy, maintaining mitochondrial quality control. (F) Healthy mitochondria are retained and trafficked along microtubules via dynein motors toward the leading edge of the cell. (G) At the invasive front (lamellipodia), polarized mitochondrial positioning ensures local ATP and ROS supply, facilitating actin remodeling, focal adhesion turnover, and directional migration.

Mitochondrial dynamics and trafficking provide a spatiotemporal layer of control for metastatic progression, not merely a change in organelle shape (Figure 3). Recent syntheses and supporting studies coalesce around a common principle: the mitochondrial fission–fusion balance serves as a spatiotemporal allocation system that matches organelle shape and positioning to the shifting bioenergetic and redox demands faced during migration and subsequent colonization. A fission‐forward state generates smaller mitochondrial units that can be redistributed toward protrusive zones, enabling localized ATP and ROS delivery to support actin remodeling and pericellular matrix remodeling, whereas a fusion‐forward state maintains elongated, continuous networks to stabilize respiratory competence and meet the enduring ATP burden of metastatic outgrowth [71, 72].

Anchored in this allocation perspective (Figure 3), the balance is established by phosphorylation‐controlled “switches” on dynamin‐related protein 1 (Drp1): extracellular signal‐regulated kinases 1/2 (ERK1/2) and cyclin‐dependent kinases 1/5 (CDK1/5) promote Drp1 activity, whereas protein kinase A (PKA) dampens it, coupling growth‐ and matrix‐derived inputs to organelle remodeling. When Drp1 is prioritized, smaller mitochondria become more readily trafficked along microtubules to lamellipodia/invadopodia, enriching these fronts with localized energetic and redox resources for protrusion function. By contrast, mitofusin 1/2 (MFN1/2) and optic atrophy 1 (OPA1) preserve a fused reticulum that secures respiratory stability for sustained outgrowth, while mitophagy culls damaged units to preserve a functionally fit pool [37]. In this regard, fission functions as a QC‐to‐deployment step: fragmentation channels low‐mitochondrial membrane potential (ΔΨm) pieces into mitophagic turnover, thereby enriching a functionally filtered pool that can subsequently be delivered to high‐demand protrusions. In line with this logic, inhibiting Drp1 or the mitochondrial transport machinery suppresses invadopodia formation and reduces metastatic burden in vivo [37].

Representative examples highlight how mitochondrial remodeling can exert bidirectional effects on invasion. In a pro‐invasive configuration, upstream transcriptional and inflammatory signals converge on Drp1 to raise fission capacity and translate organelle reshaping into motility outputs: ETS proto‐oncogene 1, transcription factor (ETS1) drives up Drp1 expression, while IL‐6 engages ERK1/2 to activate Drp1 via phosphorylation. The resulting rise in fragmentation promotes redox‐linked signaling that bolsters EMT–TF circuits and raises matrix metalloproteinase (MMP) activity, jointly enabling protrusion‐driven invasion and metastatic spread [73, 74]. Conversely, a fusion‐biased state can be pro‐metastatic: MFN1/OPA1‐driven networking sustains elevated ΔΨm and OXPHOS while spatially confining oxidant output, enabling polarized ATP/redox “hotspots” that support persistent cytoskeletal work and distal outgrowth; this association recurs across distinct tumor models [75, 76, 77]. Viewed together, these findings support a quality triage and targeted delivery model: depolarized fragments are cleared, and the remaining mitochondria are selectively routed to sites of highest workload (Figure 3). A division‐and‐forward‐transport bias is inclined to support edge‐associated invasion, whereas network reinforcement favors endurance and colonization by sustaining oxidative capacity while keeping oxidant cues spatially restricted. Since these patterns are frequently imposed by physical wiring—adhesive load paths, cytoskeletal tension, and ER–mitochondria contact dynamics—Section 2.4 addresses how biomechanical cues are transduced into mitochondrial Ca^2^^+^ routing and microdomain signaling that gate EMT‐associated migration programs.

### 2.4 Coupling Biophysical Cues to Mitochondrial Ca2+ Dynamics in Metastatic Migration

Mitochondrial mechanobiology can be framed as a context‐dependent gating system in which ECM stiffness, fluid shear, and spatial confinement tune a shared mitochondrial output triad—ΔΨm, spatially confined mtROS microdomains, and ATP availability—setting the kinetics of focal‐adhesion turnover, invadopodia‐driven matrix remodeling, and directed migration (Table 2). Recent observations indicate that stiffness does not impose a fixed fission–fusion rule; instead, outcomes depend on cue strength/duration, adhesome wiring, subcellular locale, and metabolic state. Under acute or robust rigidity inputs, mitochondria are driven toward a fission‐forward state via ROCK‐dependent Drp1 recruitment and Piezo‐type mechanosensitive ion channel component 1 (PIEZO1)—a stretch‐activated Ca^2^^+^ entry route—that activates ERK1/2 to promote Drp1 Ser616 phosphorylation. This Drp1‐skewed state restricts mtROS to localized domains and engages NF‐κB–MMP programs, in this way driving invasive behavior [78, 79]. Conversely, under prolonged mechanical loading, reassembly of the β1‐integrin adhesome can enlist particularly interesting new cysteine–histidine rich protein 1 (PINCH1)—an integrin‐adaptor that orchestrates downstream signaling—to restrain Drp1 and favor mitochondrial elongation with higher ΔΨm and sustained OXPHOS. This supports an adhesome “branching” logic: β1–kindlin‐2 coupling drives a rapid ROCK–Drp1 fragmentation burst, whereas a β1–PINCH1 module dampens Drp1 to uphold a fused, respiration‐supportive network even under matched stiffness conditions [80, 81, 82]. Drawn on this adhesome‐to‐mitochondria branching logic, actin scaffolds at endoplasmic reticulum (ER)–mitochondria contact sites can drive fission‐machinery assembly, placing mitochondria‐associated membranes (MAMs) as a cytoskeleton‐linked relay that converts mechanical stress into organelle remodeling [83, 84].

**TABLE 2:** Mechanically induced mitochondrial remodeling and its impact on cancer cell invasion across distinct biophysical cues.

Mechanical cue	Effect on mitochondrial dynamics	Structural/Functional outcome	Impact on invasive migration	Reference
ECM stiffness	Context‐dependent: acute/high‐gain stiffness → Drp1–Ser616 fission (RhoA/ROCK–ERK1/2); prolonged/β1–PINCH1 → Drp1 restraint and fusion with sustained ΔΨm/OXPHOS.	Under fission: fragmented mitochondria generate localized mtROS microdomains that couple to invadopodia. Under fusion: elongated networks elevate ΔΨm and respiratory efficiency for sustained motility.	Under acute/high stiffness (fission): invadopodia assembly via NF‐κB/MMP and accelerated invasion. Under prolonged/high‐load (fusion): sustained migration with high ΔΨm/OXPHOS.	[78, 79, 80, 81, 82, 83, 84]
Laminar shear stress	Promotes fusion; upregulates MFN2 and OPA1; downregulates Fis1; shifts Drp1 phosphorylation (Ser637↑/Ser616↓) to inhibit Drp1 recruitment.	Interconnected mitochondrial networks; elevated ΔΨm and ATP output.	Facilitates directional migration, focal‐adhesion cycling, and sustained motility under flow.	[78, 79, 85, 86]
Disturbed shear stress	Promotes fission; increases Drp1 activation (Ser616) and elevates ROS.	Fragmented mitochondria; elevated ROS; altered cristae.	Drives ROS‐dependent inflammatory signaling and EMT; may transiently enhance motility but risks cytotoxicity when ROS is excessive.	[78, 79, 85, 86]
Spatial confinement	Favors high‐ΔΨm elongated networks with rearward mitochondrial polarization; dynamic fission–fusion remodeling is required to maintain confinement‐dependent polarity.	Fused, high‐ΔΨm networks; rearward mitochondria; OXPHOS‐derived ATP supports bleb‐based motility through tight spaces.	Enables efficient passage through narrow channels; disrupting fusion–fission balance (e.g., OPA1↑ or dominant‐negative Drp1K38A) impairs confined migration.	[90, 91, 92, 93]

Shear forces can act as a flow‐pattern switch for mitochondrial remodeling: laminar shear tends to bias toward MFN2/OPA1‐supported networking with elevated ΔΨm and ATP production, whereas disturbed flow shifts the balance toward Drp1 engagement, fragmentation, and a more pro‐inflammatory oxidant‐signaling state [85, 86]. Beyond gross mitochondrial network architecture, mechanical cues propagated via the cytoskeleton and ER–mitochondria contacts can remodel cristae and inner‐membrane organization, creating localized ΔΨm/ROS microdomains (i.e., spatially restricted regions of elevated/altered ΔΨm/ROS) relevant to focal ECM degradation and migration [87], with additional shear‐dependent bioenergetic and signaling adaptations reported across models [88, 89]. Spatial restriction adds a second mechanical layer to this shear‐tuned mitochondrial logic: in narrow tracks or microchannels, cells can upshift ΔΨm and oxidative gene programs while simultaneously repositioning mitochondria to match the prevailing force‐production mode—for example, enriching rearward pools that sustain bleb‐based motility—thus linking constrained geometry to both metabolic state and subcellular energy/redox distribution [90, 91]. In this geometry‐driven setting, mitochondrial dynamics become a polarity gate: disrupting fission–fusion control blunts confinement‐locked front–rear organization and weakens invasive migration, yet cells can retain an adhesion‐imprinted “bioenergetic memory” that sustains polarized output even after exiting confinement [92, 93].

**FIGURE 4:**
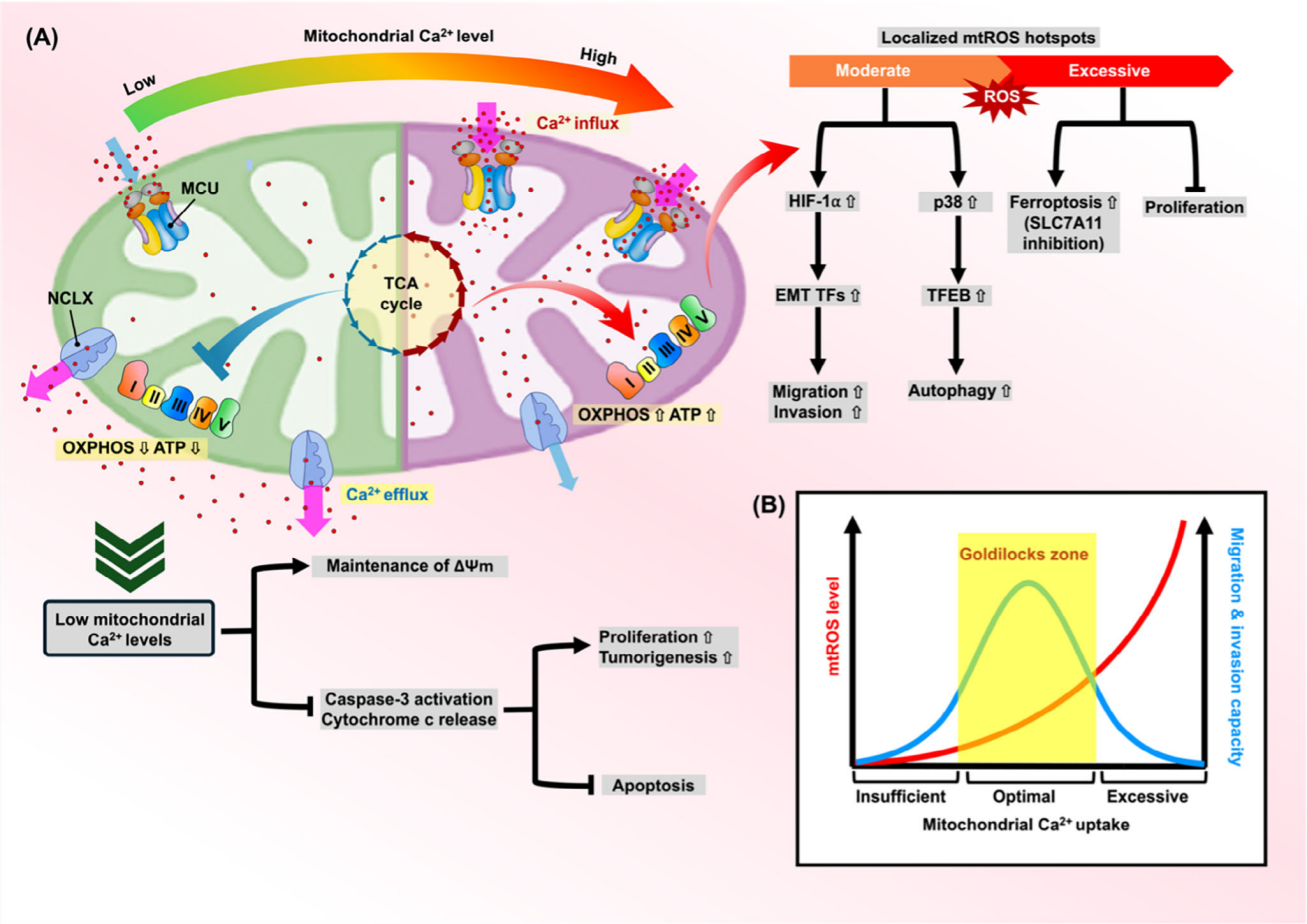
Mitochondrial Ca^2^^+^ levels determine the balance between redox signaling, energy metabolism, and metastatic fate in a dose‐dependent manner. (A) Left: Low mitochondrial Ca^2^^+^ levels impair OXPHOS, reduce ATP production, and blunt apoptosis via suppressed ΔΨm loss and cytochrome c release—promoting tumor cell survival. Right: High Ca^2^^+^ influx via the MCU boosts TCA activity, elevates ATP and mtROS generation, and promotes HIF‐1α stabilization, EMT transcription factor (TF) induction, and migration. Excessive accumulation, especially when NCLX‐mediated efflux is impaired, leads to toxic mtROS, ferroptosis, or growth suppression. (B) The "Goldilocks zone" illustrates the non‐linear relationship between mitochondrial Ca^2^^+^ uptake, mtROS levels (red curve), and migration/invasion capacity (blue curve). Both insufficient and excessive Ca^2^^+^ flux are detrimental; only intermediate uptake sustains metastatic signaling.

Alongside mechanics‐driven remodeling, mitochondrial Ca^2^^+^ flux constitutes an “ionic mechanobiology” layer that converts force‐sensitive ion flux into mitochondrial bioenergetic and redox outputs, supporting metabolic adaptability and invasive competence. Figure 4A captures a Ca^2^^+^‐to‐redox tuning axis: moderate uptake via the mitochondrial calcium uniporter (MCU) boosts TCA‐cycle dehydrogenase activity and OXPHOS, generating localized mtROS hotspots that stabilize HIF‐1α, elevate EMT transcription factors, and support motility, while also engaging a p38–TFEB arm that increases autophagy capacity. When Ca^2^^+^ efflux through the mitochondrial Na^+^/Ca^2^^+^/Li^+^ exchanger (NCLX) is impaired, mitochondrial Ca^2^^+^ rises and the circuit shifts into an excessive‐oxidant regime, linking SLC7A11 inhibition to ferroptotic vulnerability and proliferative restraint [34, 94, 95]. Altogether these findings define a mitochondrial Ca^2^^+^ “Goldilocks zone” (Figure 4B), where Ca^2^^+^ routing is tuned to support pro‐metastatic signaling without triggering cytotoxic pressure [34]. Across histotypes, recent observations converge on a unifying view in which MCU functions as a context‐sensitive Ca^2^^+^ entry valve that repartitions three coupled outputs—redox buffering (e.g., Kelch‐like ECH‐associated protein 1 (KEAP1)–Nrf2 wiring), proteostress/autophagy capacity (via a p38 mitogen‐activated protein kinase (p38 MAPK)–transcription factor EB (TFEB) arm), and cell‐fate liability (apoptotic priming vs. ferroptosis sensitivity under cystine/SLC7A11 constraint)—tuning whether Ca^2^^+^‐driven mitochondrial signaling is converted into pro‐dissemination behavior or exposed as a stress‐amplified vulnerability [96, 97, 98]. In addition to Ca^2^^+^, mitochondria also integrate K^+^, Na^+^, and Cu^2^^+^ fluxes that stabilize ΔΨm and shape redox bursts relevant to anoikis resistance and invasion; for broader coverage, see Fnu and Weber [99]; and Box 1. In aggregate, the studies reviewed here converge on a single organizing principle: biophysical forces and ion flux act in concert to “set” mitochondrial architecture, spatial deployment, and Ca^2^^+^‐linked redox output, as a result supporting invasive motility while keeping cells near a stress‐limited viability limit. Set within this mechano–ionic backdrop, Subsection 2.5 addresses the role of mitochondrial genome integrity and organelle stress‐response programs in steering metastatic fitness toward preserved OXPHOS competence or toward progressive functional decline during dissemination and outgrowth.

### 2.5 Mitochondrial Genome Integrity and Stress Response in Metastatic Spread

Clinically, alterations in mtDNA abundance and sequence fidelity in primary tumors have been correlated with aggressive behavior and adverse outcomes in specific malignancies. For example, reduced mtDNA copy number is inversely linked to unfavorable prognosis in adrenocortical and chromophobe renal cancers [100]. At the sequence‐fidelity layer, mtDNA point mutations targeting ETC genes can shift redox tone and metabolic adaptability in ways that favor EMT programs and invasion under defined constraints [7]. Since mtDNA is highly abundant and becomes mobilized during mitochondrial stress, dysfunction, and turnover, these intratumoral states can generate an extracellular “readout”: mtDNA enters the circulation via passive release (e.g., apoptosis/necrosis) as well as active trafficking routes including vesicle‐encapsulated forms [101, 102, 103]. Consistent with this premise, blood‐borne cell‐free mtDNA is being explored as a minimally invasive biomarker with longitudinal prognostic value, with reported links to tumor‐load dynamics, recurrence, and survival [104].

At the mechanistic level, recent work resolves a limited set of recurring routes by which mtDNA variation is rendered as either pro‐dissemination potential or strict constraints. First, quantitative and compositional shifts—spanning copy‐number gains and heteroplasmic lesions—can recalibrate OXPHOS flux and mtROS‐linked cytoskeletal remodeling, favoring migration and invasion in permissive settings [105, 106]. Second, when pathogenic variants become functionally catastrophic (or approach homoplasmy), ETC performance can collapse and enforce a ceiling on spread by restricting vascular entry and reducing circulating tumor‐cell yield [64]. Third, mtDNA integrity is itself plastic and can be reprogrammed by higher‐order regulators, including epigenetic remodeling and oncometabolite‐driven defects in the replication/repair machinery (e.g., fumarate‐mediated modification of DNA polymerase γ), which together accelerate respiratory erosion and mutational accrual linked to metastatic progression [107, 108]. Viewed together, these findings identify mtDNA as a dynamic variable that can either secure respiratory competence for invasion or trigger redox/respiratory failure once tolerance limits are exceeded.

Beyond cell‐intrinsic mtDNA states, tumors can modify bioenergetic capacity in trans by acquiring exogenous mitochondria or mtDNA from stromal neighbors. Mitochondrial transfer via tunneling nanotubes can rejuvenate respiration in OXPHOS‐impaired cells and enable recurrence after dormancy [109, 110]. Likewise, oxidant‐stressed stromal fibroblast populations can load mtDNA into secreted vesicular carriers through mitophagy, restoring OXPHOS in respiration‐impaired lung tumor cells and accelerating invasion and colonization [111]. Together, these observations support a trans‐acting “mitochondrial economy” in which the microenvironment functions as a distributable reservoir of mitochondrial material that reshapes metastatic competence through two coupled outputs. One output is bioenergetic rescue, whereby imported mitochondrial content reinstates oxidative capacity and sustains biosynthetic demand in otherwise respiration‐limited tumor cells [111, 112]. A second output is microenvironmental remodeling, since stress‐released extracellular mtDNA can serve as a danger‐like immune cue that activates DNA‐sensing innate pathways and consolidates myeloid‐driven immunosuppression, favoring a permissive metastatic milieu [113].

Conceptually, mtDNA status sets baseline respiratory/redox headroom, whereas stress‐response programs determine whether that reserve is buffered or breached during metastatic challenge. Alongside this layer, the mtUPR offers a complementary adaptation axis that links mitochondrial proteostasis to dissemination: proteotoxic and oxidative cues engage activating transcription factor (ATF) family signaling (e.g., ATF4/ATF5) and C/EBP homologous protein (CHOP), driving nuclear induction of chaperone modules such as heat shock protein (HSP) family factors (e.g., HSP60/HSP10) and proteolytic circuits exemplified by caseinolytic mitochondrial matrix peptidase proteolytic subunit (ClpP) and lon peptidase 1, mitochondrial (LONP1), while tuning mitophagy [35]. By refolding or removing damaged proteins, coupling selective turnover to biogenesis control, and modulating antioxidant buffering, mtUPR sustains ΔΨm and respiratory flux while restraining runaway ROS [114]. In cancer settings, a hormetic mtUPR window can enhance sirtuin 3 (SIRT3)–forkhead box O3 (FOXO3)–linked antioxidant capacity and cytoskeletal remodeling, priming migratory behavior [115], whereas sustained mtUPR signaling can engage Wnt/β‐catenin and HIF‐1α programs that promote EMT and metastatic establishment [35]. To be metastasis‐relevant, mtUPR‐driven proteostasis capacity has to be synchronized with growth and energy‐stress signaling that sets metabolic throughput. Therefore, crosstalk with AKT and AMP‐activated protein kinase (AMPK) further adapts mitochondrial architecture–function for the ATP and redox demands of motile tumor cells [116]. As a whole, genome‐encoded capacity, transferable organelle resources, and stress‐adaptation circuitry together define a tunable fitness envelope that shapes how close disseminating cells operate to failure under escalating demands. With this organelle‐fitness landscape in place, Section 3 dissects how selective mitochondrial turnover—mitophagy—is programed by cell‐intrinsic programs and niche pressures to regulate EMT/MET dynamics and metastatic fitness. This framing also anticipates how ubiquitin‐driven labeling and editing mechanisms gate MQC, which we detail in Section 4.

## 3 Cell‐Autonomous and Microenvironmental Regulation of Mitophagy: Implications for EMT/MET and Metastatic Fitness

Mitophagy is a selective MQC program whose influence on cancer cell migration and metastasis is dictated by tumor‐intrinsic state, microenvironmental constraints, and disease stage—i.e., by the evolving interplay between intrinsic programs and extrinsic inputs over time [117]. Its outputs can diverge in direction: when mitochondrial ROS remain within a signaling window, mitophagic pruning can dampen pro‐dissemination cues and restrain spread; under intense oxidative burden, however, the same process becomes cytoprotective by preserving organelle integrity and averting apoptosis, in turn sustaining motile competence and metastatic persistence [118, 119]. In solid tumors, where hypoxia and nutrient limitation are pervasive, mitophagy often acts as a stress‐adaptation module linking mitochondrial maintenance to survival‐associated invasive behavior—low oxygen generally favoring persistence and distant seeding, while nutrient deprivation yields outcomes that depend on the magnitude and duration of flux [120]. Notably, amino‐acid withdrawal typically provokes a stronger response than glucose restriction; if activation overshoots, net mitochondrial depletion can destabilize cytoskeletal organization and blunt metastatic fitness [121]. Mechanical and biophysical features of the TME—including matrix stiffness, culture dimensionality, and shear forces—add another layer of control that biases when mitophagy supports dissemination versus imposes functional constraint [122]. Table 3 synthesizes these context‐conditioned associations, emphasizing the pleiotropic and occasionally paradoxical roles of mitophagy across the invasion–metastasis cascade and orienting the mechanistic subsections that follow.

**TABLE 3:** Divergent impacts of intrinsic and extrinsic mitophagy regulators on metastatic phenotypes.

Type	Category	Subcategory	Mechanism	Effect on metastasis	Reference
Intrinsic factors	mtROS Level	—	Bidirectional regulation between ROS and mitophagy; regulates EMT and metastatic behavior in a stage‐dependent manner.	Context‐dependent: suppresses early metastasis, enhances late‐stage progression.	[123]
	Epigenetic regulation	DNA Methylation	Promoter methylation of mitophagy genes (e.g., BNIP3) represses mitophagy, enhances mtROS, stabilizes HIF‐1α, and promotes EMT.	Enhances EMT and metastasis when hypermethylated; demethylation suppresses progression.	[8, 127]
		Histone Modification	Histone acetylation/methylation affects transcription of mitophagy genes (e.g., PINK1, BNIP3); HDAC inhibitors enhance Parkin‐mediated mitophagy.	Promotes mitophagy and suppresses tumor cell proliferation and migration.	[130, 131, 132, 133]
		Non‐coding RNA	lncRNAs and miRNAs modulate mitophagy via direct targeting or ceRNA mechanisms, affecting EMT‐related genes and mitochondrial clearance.	Fine‐tunes mitophagy and promotes or inhibits migration based on ncRNA expression pattern.	[137, 138, 139, 140]
		Epitranscriptomics	m6A RNA modifications (e.g., ULK1 mRNA) regulate mitophagy post‐transcriptionally, enhancing proliferation and metastasis.	Enhances mitophagy and promotes migration, invasion, and tumor growth.	[141, 142]
	Cellular Stiffness	—	Low stiffness promotes mitophagy and EMT; high stiffness may inhibit migration or enhance metastatic potential depending on context.	Reduced stiffness promotes migration and EMT through mitophagy; highly context‐specific.	[152, 153, 154, 155, 156, 157, 158]
Extrinsic cues	Nutrient deprivation	Glucose	Mild AMPK activation supports mitophagy and migration; excessive deprivation insufficient for bulk mitophagy.	Supports invasion through redox and energy balance.	[162]
		Amino acid	Robust mTOR inhibition and TFEB activation leads to excessive mitophagy, cytoskeletal collapse, and impaired metastasis.	Suppresses invasion due to excessive mitophagy and ATP depletion.	[160, 161]
	Culture dimensionality	2D vs. 3D	3D cultures show increased basal mitophagy, redox stability, and EMT; 2D cultures have limited autophagy activation.	Promotes EMT and drug resistance via basal autophagy/ mitophagy flux.	[165, 166, 167]
	Biomechanical cues	ECM stiffness	Intermediate stiffness enhances mitophagy and migration; excessive rigidity blocks motility; soft matrices increase DRP1/MIEF1 activation.	Supports or inhibits invasion depending on stiffness threshold.	[168, 170, 171]
		Shear stress	Shear stress induces AMPK and Parkin/PINK1 mitophagy; high flow selects deformable or clustered cells for metastasis.	Enables survival and extravasation; regulates cortical tension and mitophagy flux.	[172, 173]
	Hypoxia	—	Hypoxia induces BNIP3/BNIP3L mitophagy, supports EMT and invasion; normoxia maintains low basal mitophagy and suppresses EMT.	Supports pro‐invasive phenotypes under hypoxia; suppresses EMT under normoxia.	[120, 175]
	Stromal cell interaction	CAFs/CAMs	CAFs and CAMs modulate mitophagy via miRNAs, mtDNA transfer, cytokines (e.g., MIF, IL‐33), affecting EMT and metastatic fitness.	Facilitates metastatic niche establishment and mitochondrial homeostasis.	[111, 179, 180, 181]

### 3.1 Tumor‐Intrinsic Regulators of Mitophagy and Their Impact on Metastatic Behavior

**FIGURE 5:**
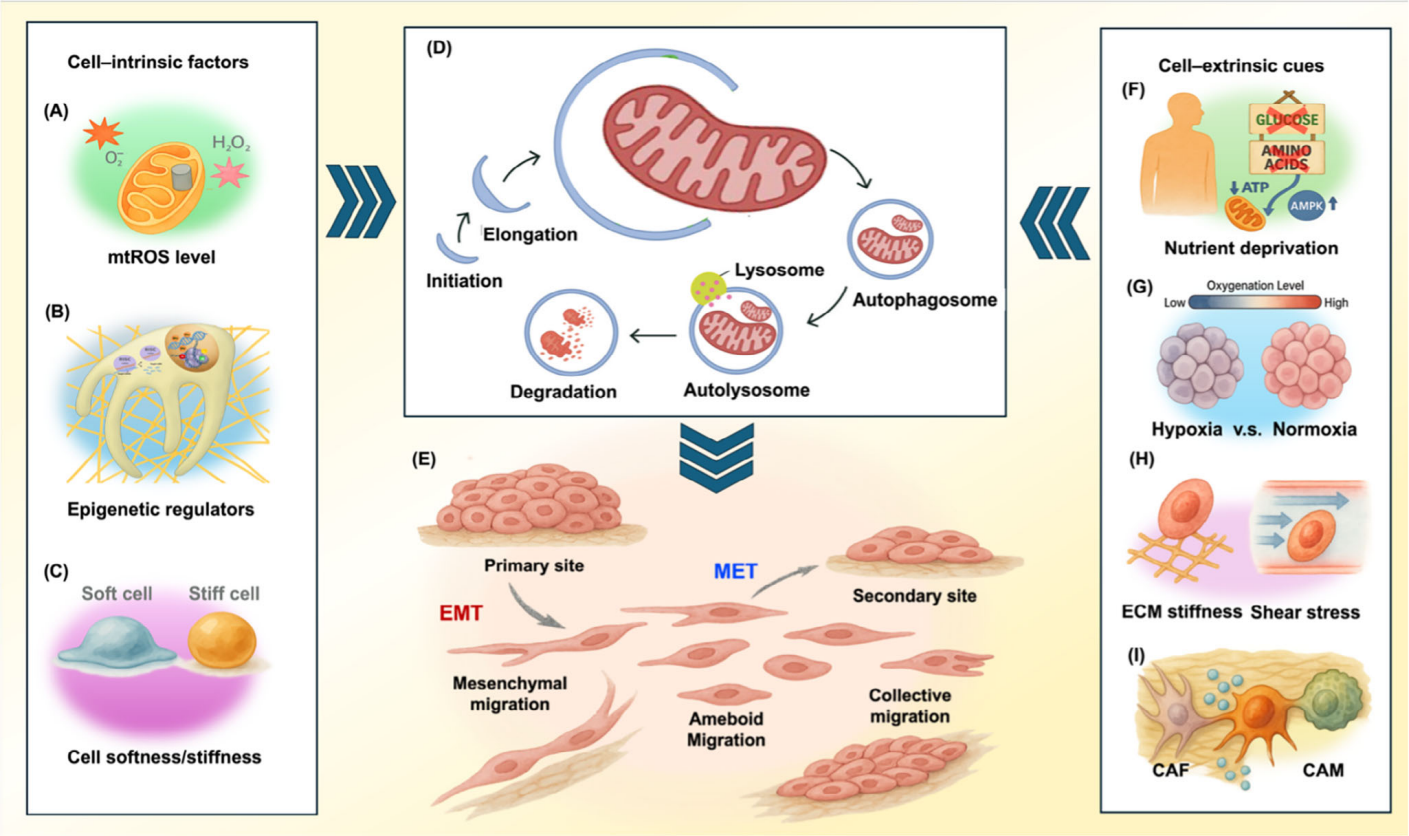
Context‐dependent regulation of mitophagy and EMT/MET plasticity by cell‐intrinsic factors and microenvironmental cues. Cell‐intrinsic drivers (left) and extrinsic stresses (right) converge on the mitophagy machinery (D) to remodel mitochondrial quality, dictating transitions along the EMT–MET spectrum (E) and the mode of tumor‐cell migration. (A) Mitochondrial ROS levels act as redox rheostats that bidirectionally tune mitophagy flux. (B) Epigenetic regulators (DNA/histone modifications, ncRNAs) fine‐tune transcription of mitophagy genes. (C) Cell softness or stiffness determines cytoskeletal tension, which feeds back on mitochondrial dynamics and clearance. (D) Canonical mitophagy cascade: initiation, phagophore elongation, autophagosome formation, fusion with lysosome, cargo degradation. (E) EMT/MET plasticity and migration modes: mesenchymal, amoeboid and collective migration underlie dissemination from the primary to secondary niche. (F) Nutrient deprivation activates AMPK‐linked mitophagy to spare energy. (G) Hypoxia versus normoxia controls HIF‐1α‐dependent mitophagy receptors. (H) Mechanical forces—namely ECM stiffness and fluid shear stress—drive stress‐tuned remodeling of mitochondrial morphology. (I) Stromal cells (e.g., CAF and CAM) secrete cytokines and metabolites that modulate tumor mitophagy and motility.

Within cancer cells, mitophagy is set by internal circuitry that steers mitochondrial performance and phenotypic plasticity. During progression, this regulation is increasingly intertwined with redox pressure, chromatin/RNA‐layer remodeling, and cell‐intrinsic mechanical properties that reshape mitochondrial performance profiles [25]. In the subsections below, we examine three intracellular levers—mtROS load, epigenetic/epitranscriptomic control of pathway capacity, and cell stiffness as a mechanotransductive input (Figure 5A–C)—and delineates how they reprogram mitophagy dynamics (Figure 5D) to reorient EMT/MET trajectories, motility, and organ‐selective outgrowth (Figure 5E). We first address mtROS–mitophagy coupling, then summarize gene‐ and RNA‐level tuning of mitophagy programs and ultimately explore stiffness‐linked mitochondrial signaling that converges on mitophagy to shape metastatic behavior.

#### 3.1.1 Mitochondrial ROS as a Contextual Modulator of Mitophagy in Metastasis

A reciprocal mtROS–mitophagy circuit can be viewed as a redox “set‐point” controller that either restrains or promotes metastatic behavior. Through targeted removal of damaged mitochondria, mitophagy keeps mitochondrial oxidant load below an inflammatory threshold and curbs stress‐linked cytokine output; when this buffering fails, ROS surges engage the NOD‐like receptor family, pyrin domain containing 3 (NLRP3) inflammasome to boost interleukin‐1β, a switch that can favor metastatic engraftment—most discernibly in breast cancer bone tropism [118]. In a feed‐forward extension of this set‐point model, crossing the inflammatory threshold can render the circuit self‐reinforcing: insufficient clearance leaves dysfunctional mitochondria in place, sustaining mtROS pulses and their downstream signaling outputs. As a result, redox‐driven programs persist longer and malignant progression can accelerate [6]. Importantly, the phenotypic “sign” of this feedback is stage‐contextualized: early in tumor evolution, mtROS‐evoked mitophagy often functions as a checkpoint on dissemination by pruning high‐ROS organelles, whereas later it can flip toward pro‐dissemination when its bioenergetic and cytoprotective benefits preferentially bolster invasion and survival [123].

Under conditions of limited energetic pressure, intermediate oxidant signaling can be transduced into a pro‐migratory program through suppression of prolyl‐hydroxylase domain (PHD) enzymes, locking in HIF‐1α stabilization and upregulating SNAI1, TWIST1, and ZEB1 [69]. Here, mitophagy dampens further escalation by clearing oxidant‐loaded mitochondria, curtailing HIF‐1α–coupled EMT reinforcement and reducing metastatic initiation [69]. With disease progression, the cytosolic redox burden mounts; excessive oxidative stress perturbs cell–cell junctional integrity, boosts MMP output to accelerate ECM breakdown, and reorganizes actin‐network architecture, jointly enhancing invasive behavior and diminishing therapeutic response [124]. Under these harsher conditions, mitochondrial turnover becomes central for organelle fidelity and bioenergetic adequacy: removal of damaged mitochondria preserves respiratory capacity and ATP supply to fuel migration and invasion, while minimizing oxidative injury yet keeping a signaling‐competent redox tone within a tolerable window [125]. Together, these observations position mitophagy as a tunable rheostat that balances mitochondria‐linked redox signaling against organelle damage, influencing whether EMT circuitry is curtailed or motile fitness is sustained as stress and energetic requirements intensify. This mechanistic schema sets up Section 3.1.2, which dissects how epigenetic/epitranscriptomic control adjusts mitophagy capacity and reactivity across shifting cellular stress states.

#### 3.1.2 Epigenetic Landscapes Shaping Mitophagy‐Driven Metastatic Behavior

Whereas Section 3.1.1 frames mtROS as an acute input that changes turnover demand, longer‐horizon differences in metastatic fitness arise when chromatin and RNA‐layer programs reset the capacity and inducibility of mitophagy nodes. Here we summarize four tiers—DNA methylation, histone modification, non‐coding RNA circuits, and epitranscriptomic marks—that jointly bias receptor‐type versus PINK1/Parkin execution and tilt EMT/MET switching under stress.

##### 3.1.2.1 DNA Methylation

DNA methylation—catalyzed by DNA methyltransferases (DNMTs) (e.g., DNMT1 and DNMT3A) —can suppress promoters of mitophagy‐related genes, including BCL2/adenovirus E1B 19 kDa‐interacting protein 3 (BNIP3), compromising receptor‐mediated mitochondrial clearance [126]. In gastric cancer cells under methionine‐restricted conditions, downregulation of long non‐coding RNA (lncRNA) PVT1 undermines its interaction with DNMT1, permitting BNIP3 promoter demethylation and restoring mitophagic flux, with coordinated restraint of proliferative and EMT‐leaning traits [127]. In contrast, BNIP3 promoter hypermethylation in low‐O_2_ conditions associates with mtROS rise, HIF‐1α stabilization, and EMT activation, supporting greater metastatic potential [8]. Beyond promoter‐mediated gating, methylation heterogeneity across the BNIP3L (NIX) locus can relocate intron‐1 occupancy from CCCTC‐binding factor (CTCF) to its paralog, Brother of the Regulator of Imprinted Sites (BORIS), producing isoform‐specific outputs that differentially govern autophagy/mitophagy routing across oxygen conditions [128]. In parallel, hypoxia‐linked DNMT3A activity can recast site‐specific methylation to drive HIF‐1α recruitment and EMT‐TF engagement, acting alongside BNIP3/BNIP3L programs to support protrusion formation and matrix remodeling [129]. Overall, these lines of evidence position DNA methylation as a receptor‐availability gate at BNIP3/BNIP3L loci, determining how effectively hypoxic signaling is translated into receptor‐type mitochondrial surveillance capacity and, in turn, into EMT‐associated invasive output.

##### 3.1.2.2 Histone Modification

Reversible histone acetylation and methylation epigenetic circuits remodel chromatin accessibility at mitophagy genes (including PINK1 and BNIP3), defining how readily these loci respond to metabolic or oxidative pressure [130]. Consistent with this capacity‐control mode, epigenetic silencing at the BNIP3 promoter dampens receptor‐mediated mitophagy, is enriched in advanced solid tumors, and—under high‐stress conditions—may bias cytoskeletal and adhesion wiring in ways that correspond to mesenchymal–amoeboid invasion plasticity [131]. Across tumor contexts, acetylation editing can tune mitophagy by acting at both the execution layer and the transcriptional layer: histone deacetylase (HDAC) inhibition prompts Parkin acetylation to upshift PINK1/Parkin‐dependent flux and counter proliferative/migratory output, whereas SIRT1‐driven deacetylation of FOXO3 promotes a mitophagy‐supportive transcriptional state that helps sustain growth under resource constraint [132, 133]. Alongside these mitophagy‐facing layers, acetylation status also feeds directly into the invasion apparatus: HDAC6‐driven cortactin deacetylation promotes invadopodia formation and matrix proteolysis, whereas HDAC6 inhibition increases cortactin acetylation and suppresses invasive motility, partly via p300 stabilization and wider chromatin remodeling [134, 135]. Mechanistically, histone/lysine acetylation connects mitophagy capacity to invasive competence by jointly tuning chromatin accessibility at mitophagy loci (e.g., PINK1/BNIP3) and the acetylation state of protrusion/ECM‐remodeling effectors. Re‐tuning across these layers by metabolic stress, hypoxia‐associated chromatin repression, or HDAC/SIRT activity can therefore redirect invasion mode instead of fixing invasion into a pro‐ or anti‐metastatic outcome. This framework provides a bridge to Section 3.1.2.3, where non‐coding RNAs introduce a rapid post‐transcriptional layer that adjusts receptor abundance and PINK1/Parkin flux to local constraints.

##### 3.1.2.3 Non‐Coding RNA–Based Post‐Transcriptional Regulation

At the post‐transcriptional tier, non‐coding RNAs (ncRNAs)—comprising lncRNAs, microRNAs (miRNAs), and circular RNAs (circRNAs)—shape mitophagy kinetics without changing genomic sequence. The lncRNA MALAT1 has been documented to localize to mitochondria and serve as a retrograde regulator, either by tuning CpG‐site methylation in mtDNA or by recruiting chromatin‐remodeling machinery that modulates PINK1/BNIP3 expression [136]. Downstream of lncRNA‐mediated regulation, miRNAs offer a second post‐transcriptional gate by setting the abundance of mitophagy receptors. miR‐137 represses FUN14 domain‐containing protein 1 (FUNDC1) and also NIP3‐like protein X (NIX; also called BNIP3‐like, BNIP3L), reducing mitophagy and limiting invasion [137]. Conversely, lncRNA_049808 flips this relationship by serving as a miRNA sponge that sequesters miR‐101, which relieve repression of FUNDC1 and enhancing mitophagy‐linked migratory output in TNBC models [138]. Beyond lncRNA–miRNA crosstalk, circRNAs provide an complementary RNA‐level regulatory axis that can shift control toward the PINK1/Parkin arm: m5C‐modified circRREB1 activates heat shock protein family A (Hsp70) member 8 (HSPA8), as such engaging PINK1/Parkin‐dependent mitochondrial turnover and driving tumor progression in lung cancer settings [139]. In patient‐cohort transcriptomic profiling of ovarian cancer, mitophagy‐associated lncRNA/ceRNA signatures correlate with prognosis and therapy response, supporting a clinically observable layer of ncRNA‐based control [140]. In sum, ncRNA networks constitute a rapid RNA‐level routing layer that reallocates control between receptor‐mediated entry points and PINK1/Parkin execution, translating mitochondrial stress handling into adaptable invasive outputs; Section 3.1.2.4 extends this logic to epitranscriptomic marks that tune transcript fate to further set mitophagy capacity and responsiveness.

##### 3.1.2.4 Epitranscriptomic Regulation

RNA chemical modifications insert an extra layer of control over mitophagy by reshaping transcript stability and translation of key regulators. In epithelial ovarian cancer, Wilms tumor 1–associating protein (WTAP) installs N6‐methyladenosine (m6A) within the 5′ untranslated region (5′‐UTR) of unc‐51‐like kinase 1 (ULK1) mRNA; this mark is read by insulin‐like growth factor 2 mRNA‐binding protein 3 (IGF2BP3), which stabilizes the transcript and promotes kinase‐dependent mitophagy, accordingly fueling growth and migration [141]. In small‐cell lung cancer, methyltransferase‐like 3 (METTL3) installs N6‐methyladenosine (m6A) on decapping mRNA 2 (DCP2) transcripts, suppressing DCP2 decapping activity and reinforcing PINK1/Parkin‐mediated mitophagy and chemoresistance; treatment with the small‐molecule METTL3 inhibitor STM2457 reverses these effects [142]. Beyond m6A, mitochondrial tRNA modification networks can couple translation to QC by setting how effectively cells maintain mitochondrial proteostasis and engage mitophagy under stress. Against this framework, impairment of tRNA editing hampers mitophagy‐dependent organelle turnover, whereas m7G–tRNA–driven translational remodeling can redirect signaling toward mechanistic target of rapamycin (mTOR), a central nutrient‐sensing kinase, and its RPTOR‐containing mTOR complex 1 (mTORC1), imposing downstream brakes on ULK1‐mediated initiation and retuning mitophagy flux [143, 144]. Overall, epitranscriptomic regulation acts as a quantitative throttle on mitochondrial turnover by altering the net abundance and activity of mitophagy pathway sentinels—both through mRNA fate decisions that potentiate initiation/execution and through tRNA‐linked translation‐state rewiring that interfaces with mTOR signaling—yielding druggable routes to fine‐tune stress tolerance and dissemination in a setting‐dependent manner. With this RNA‐layer “capacity setting” established, Section 3.1.3 turns to cell‐intrinsic mechanics (stiffness) as a co‐determinant that can hit shared upstream nodes to retune flux dynamics and metastatic behavior.

#### 3.1.3 Intrinsic Cellular Stiffness as a Potential Biomechanical Cue for Mitophagy‐Driven EMT

Intrinsic cellular stiffness reflects the coupled mechanics of the plasma membrane, cytoskeleton, and nucleus. The perinuclear cytoskeleton can buffer strain and shape nuclear deformation, while contractile‐force transmission via the linker of nucleoskeleton and cytoskeleton (LINC) complex raises the effective stiffness of the nucleus and remodels chromatin organization [145, 146]. In a broad range of cancers, greater deformability can facilitate passage through confined interstitial spaces during dissemination, but the direction and magnitude of rigidity remodeling vary with lineage and niche, mirroring context‐dependent reshaping of actomyosin contractility, RhoA/ROCK signaling, and nucleus–cytoskeleton coupling [147, 148, 149]. Clinically relevant cases include HCC subpopulations in which reduced stiffness tracks with more robust motility through actomyosin and c‐Jun N‐terminal kinase (JNK) signaling [150], and endothelial co‐culture conditions where invasive breast cancer cells soften microvascular monolayers and promote transendothelial migration [151].

A frequent—yet non‐universal—pattern is an inverse link between stiffness and aggressiveness: reduced stiffness can track with enhanced migration and invasion in breast and cervical cancer lines [152], whereas highly metastatic prostate PC3 cells have been reported to be stiffer than less aggressive counterparts, highlighting tissue‐specific mechanical logic [153]. These ostensibly discordant trends become more interpretable if stiffness is treated as an upstream constraint that reweights cytoskeletal load paths and, in parallel, reshapes mitochondrial positioning, dynamics, and metabolic output—parameters that condition the stress signals (oxidant tone, Ca^2^^+^ handling, ΔΨm) that gate selective mitochondrial turnover. By routing force through actin and microtubules, cells can reshape mitochondrial fission–fusion and ATP supply, which in turn reweights oxidant tone, Ca^2^^+^ handling, and ΔΨm—an integrated signaling triad that dictates mitophagy engagement and EMT‐associated responses [154, 155]. In keeping with this reciprocity, interventions that reweight mitochondrial oxidative burden and organelle abundance can reduce invasion‐linked phenotypes; in an opposite pattern, mechanically compliant fractions carry heightened tumorigenic/stem‐like wiring, and fusion disruption (MFN2 loss) increases stiffness with reduced motility—jointly pointing to a mitochondria–mechanics feedback that tunes dissemination rather than a one‐way hierarchy [156, 157, 158]. As a whole, current evidence reinforces a mechanistic model wherein a cell's intrinsic mechanical phenotype defines the operating range of mitochondria‐derived stress cues and the activation threshold for selective organelle turnover, in turn biasing EMT‐linked locomotor programs under confinement and mechanical load. Section 3.2 shifts to tumor‐extrinsic metabolic, hypoxic, and biophysical pressures that reset this shared axis across the invasion–metastasis cascade.

### 3.2 Microenvironmental Cues Governing Mitophagy: Implications for EMT/MET Transitions in Cancer

Within the TME, physical, chemical, and paracrine signals intersect at mitophagy to re‐equilibrate mitochondrial fitness, redox balance, and ATP supply, shaping epithelial–mesenchymal plasticity across the invasion–metastasis cascade. Here, “context” can be operationally resolved into four interlinked axes—nutrient availability, oxygen tension, tissue geometry/mechanics, and stromal signaling—that together set mitophagy mode (PINK1/Parkin versus receptor‐driven), magnitude, and lysosomal throughput, ultimately shifting the balance between EMT‐like migratory outgrowth and MET‐leaning re‐epithelialization. Periodic nutrient stress can split mitophagy into divergent trajectories (Figure 5F): upstream signaling may prioritize a PINK1/Parkin‐dependent response that maintains motile capacity or activate receptor‐clearance amplification that erodes mitochondrial content, drains ATP, and limits invasion. Oxygen gradients further partition pathway use (Figure 5G), with hypoxic cores favoring BNIP3/BNIP3L‐linked routes while normoxic regions maintain a low basal flux that mainly prunes stress signaling. Mechanics adds another tuning dial (Figure 5H): switching from rigid 2D to compliant fibrillar 3D matrices, in concert with stiffness and interstitial‐shear gradients, recasts fragmentation–clearance coupling and the EMT/MET balance. Microenvironmental conditioning can then lock in these states (Figure 5I), as extracellular vesicles (EVs), cytokines, and metabolites from fibroblasts‐ and macrophage‐lineage stromal cells rewire TFEB‐, PINK1‐, mTOR‐centered signaling to fortify dissemination‐permissive niches. In practice, the phenotypic outcome hinges on whether mitochondrial turnover stays homeostatic or outstrips cellular capacity, pushing cells toward motility retention or functional decline. Nutrient deprivation offers a tractable context in which this balance diverges, and Section 3.2.1 on this basis contrasts glucose restriction with amino‐acid starvation to clarify how distinct upstream programs shape mitophagy outputs.

#### 3.2.1 Glucose and Amino Acid Starvation: Contextual Activators of Mitophagy in EMT Control

Owing to impaired angiogenesis, metabolic competition, and high energetic demand, tumor cells frequently experience nutrient limitation, a pressure that retasks autophagy/mitophagy to sustain viability and shape metastatic behavior; glucose and amino‐acid deprivation are among the most prevalent stressors [159]. Amino‐acid scarcity more potently activates the lysosome–autophagy transcriptional axis by suppressing mTORC1 and promoting TFEB nuclear entry in two waves—an initial calcineurin‐dependent burst followed by sustained dephosphorylation while mTORC1 remains inactive—thus inducing autophagy/mitophagy genes within the coordinated lysosomal expression and regulation (CLEAR) network [160]. Under nutrient‐replete conditions, an upstream stimulatory factor 2 (USF2)–HDAC1 repressor complex limits chromatin accessibility at CLEAR motifs, reducing TFEB‐driven transcription and helping explain the broader lysosomal–autophagy response to amino‐acid withdrawal [161].

By contrast, glucose limitation more frequently activates an AMPK–ULK1 axis that sustains basal autophagy and can recruit a phosphoinositide kinase, FYVE‐type zinc finger containing (PIKFYVE) pathway to generate phosphatidylinositol 5‐phosphate (PtdIns(5)P)–enriched autophagosomes, typically yielding a weaker and more transient flux than amino‐acid deprivation [162]. Functionally, this metabolic split is mirrored at the phenotype level: glucose withdrawal tends to keep turnover in a homeostatic range that preserves energy/redox balance compatible with movement, whereas amino‐acid starvation more readily triggers high‐amplitude, receptor‐linked clearance episodes that can overwhelm cellular buffering [162]. A clinically relevant illustration comes from methionine restriction in gastric cancer, which dismantles a DNMT1‐centered repressive complex, diminishes BNIP3 promoter methylation, and reactivates BNIP3—driving robust mitophagy, fragmentation, ATP loss, and reduced migration [127]. A comparable stress‐sensitization principle translates to gynecologic tumors, where combining amino‐acid pressure with OXPHOS inhibition or curtailed mitochondrial biogenesis further potentiates receptor‐mediated clearance and inhibits invasion, revealing a context‐dependent vulnerability [163]. As a whole, glucose restriction and amino‐acid withdrawal place mitophagy into two distinct operating regimes—one that stabilizes mitochondrial performance under energy stress, and another that ramps up organelle turnover when lysosomal biogenesis programs prevail, with separable outcomes for EMT‐state maintenance. Importantly, these regimes are not fixed: culture geometry and tissue mechanics can recalibrate mitochondrial damage load and clearance efficiency, providing the rationale for Section 3.2.2 on dimensionality‐ and force‐dependent tuning.

#### 3.2.2 Culture Dimensionality and Mechanical Forces Shape Mitophagy‐Mediated EMT/MET Plasticity and Invasive Potential

Comparisons of 2D monolayers and 3D ECM‐based cultures robustly demonstrate that invasion programs diverge, since 3D migration requires cells to negotiate fiber confinement, variable ligand access, adhesion remodeling, and often MMP‐driven matrix reorganization [164]. In 3D matrices, invasion can also show a biphasic dependence on stiffness, with intermediate rigidity maximizing contractility‐driven movement while excessive stiffness restricts motility—an effect less apparent on rigid 2D substrates [165]. In parallel with the distinct physical demands of 3D invasion—where confinement, ligand heterogeneity, adhesion turnover, and stiffness‐dependent contractility jointly set the migratory mode—3D tissue geometry also reweights mitochondrial quality‐control circuitry. Across spheroid settings, these constraints often intensify stress‐adaptive autophagy/mitophagy engagement while making pathway completion contingent on downstream lysosomal throughput, forging a joint “signal–capacity” logic in which survival and invasion‐relevant fitness are bolstered, yet lysosomal processing and core autophagy nodes can become actionable bottlenecks under added acute challenge (e.g., therapy) [166, 167]. Viewed together, 3D mechanical architecture couples migratory decision‐making to mitochondrial maintenance by elevating QC demand while exposing lysosomal degradative capacity as the bottleneck that therapy can unmask.

Across the metastatic cascade, mechanical cues act as sequential filters that first prime invasive programs in solid tissues and then select circulation‐competent states under flow. At the primary tumor site, ECM stiffening augments adhesion–traction signaling (integrin/FAK with Rho–ROCK engagement), weakening junctional restraint and facilitating EMT‐linked transcriptional programs that support 3D invasion and metastatic outgrowth [168]. Subsequent to intravasation, interstitial and hemodynamic shear repositions the dominant constraint from traction to cortical mechanics, engaging mechanosensitive Ca^2^^+^ signaling and YAP/TAZ‐linked transcriptional outputs alongside RhoA/ROCK‐driven actomyosin remodeling; these programs can select against vulnerable single cells while enriching deformable single‐cell variants or collective assemblies that better tolerate shear and enhance subsequent seeding efficiency [169]. Having outlined how stiffness and flow impose sequential selection pressures, we next focus on the mechanistic conduit—how matrix and shear cues couple to mitochondrial remodeling and quality‐control pathways, including bulk autophagy and selective mitophagy.

Mechanistically, biophysical cues can engage both bulk autophagy and selective mitophagy: rigidity can elevate autophagic flux in stromal fibroblasts, whereas softer matrices promote peri‐mitochondrial F‐actin and Drp1/MIEF1‐dependent fission, altering mitochondrial dynamics and redox state [170, 171]. Consistent with a mechanics‐to‐mitophagy link in cancer, substrate compliance can enhance ER–mitochondria Ca^2^^+^ crosstalk, drive fission, and activate PINK1/Parkin‐mediated mitophagy [172], and interstitial flow can engage mitochondrial AMPK to increase mitophagy and bioenergetic resilience, with pathway inhibition attenuating flow‐induced motility [173]. Overall, physical context governs whether mitochondrial clearance is resolving or self‐limiting, accordingly shaping EMT/MET reversible programs via its downstream metabolic and signaling consequences. This demand–capacity view of mechanoregulated autophagy has been broadly synthesized in recent reviews of autophagy–mechanics coupling, offering a conceptual framework to link stiffness/flow cues to mitochondrial remodeling [174]. Since oxygen tension re‐scales the same logic articulated above—by altering mitochondrial insult pressure and clearance throughput—the next subsection contrasts hypoxia with normoxia to explain how mitophagy is rewired to tune invasive phenotype.

#### 3.2.3 From Hypoxia to Normoxia: Shifting Mitophagic Modes to Fine‐Tune EMT and Invasive Phenotype

In solid tumors, perivascular regions tend to remain relatively normoxic whereas poorly perfused cores persist in hypoxia, establishing oxygen gradients that reshape mitophagy and metastatic behavior. In a low‐oxygen context, HIF‐1α stimulates transcriptional upregulation of mitophagy receptors—particularly BNIP3 and BNIP3L—in doing so enhancing mitochondrial clearance to preserve bioenergetic competence and redox balance compatible with invasion [175]. As one illustration of this receptor‐linked program, oral squamous cancer stem cells subjected to 1% O_2_ display BNIP3‐requiring mitochondrial turnover, coincident with faster spheroid growth, improved scratch closure, and heightened invasiveness—effects attenuated when BNIP3 is silenced [176]. In comparison, in normoxic regions, mitophagy operates at a lower steady‐state rate—often via PINK1/Parkin—and primarily buffers transient ROS surges rather than amplifying energy output. At this baseline, mitophagy can function as signal‐pruning QC that restrains inappropriate EMT initiation by limiting stress‐linked signaling [120]. Accordingly, oxygen gradients reorient the entry route and regulatory wiring of mitochondrial clearance—hypoxia preferentially engaging HIF‐1α–responsive receptor programs, while normoxic regions favor low‐flux surveillance that limits stress signaling. The mechanistic foundation for hypoxia–normoxia switching of mitophagy programs— including (i) receptor activation logic and (ii) ubiquitin‐dependent modulation at the HIF‐1α and ER–mitochondria contact‐site levels, is dissected in Section 4.3 (especially 4.3.1–4.3.3). With this oxygen‐dependent “routing” in place, the next subsection turns to how stromal cells further reshape mitophagy in both compartments to condition EMT/MET behavior and metastatic spread.

#### 3.2.4 Mitophagy at the Stromal Frontier: How Fibroblasts and Macrophages Regulate EMT and Metastatic Spread

Cancer‐associated fibroblast (CAFs) and cancer‐associated macrophages (CAMs) are principal TME effectors whose mitophagy‐linked functional states can influence tumor‐cell motility, invasion, and metastatic fitness [177]. CAFs are a heterogeneous stromal population shaped by reciprocal tumor–stroma crosstalk that orchestrates matrix remodeling, immune conditioning and cytokine production; their accumulation is associated with poor prognosis and treatment resistance [178]. Beyond these classical functions, a biphasic EV‐driven circuit illustrates how CAF programming can couple to mitochondrial upkeep: tumor‐derived EVs from highly metastatic lung cancer cells deliver miR‐1290 to fibroblasts, suppress mitochondrial GTPase 1 (MTG1), activate AKT, and—along with ambient ROS—raise autophagy/mitophagy and promote CAF conversion; mitophagy‐high CAFs then release EVs carrying mtDNA fragments that tumor cells uptake to compensate mitochondrial genome defects, reinstate respiratory capacity, increase oxidative‐stress tolerance, and enhance invasion and metastasis [111].

CAMs likewise modulate mitophagy to shape EMT and dissemination. CAM‐derived signals can suppress or rewire PINK1/Parkin and TFEB–lysosomal programs, altering ROS, metabolism, and cytokine outputs that feed back onto tumor‐cell plasticity and invasiveness. For example, CAM‐derived macrophage migration inhibitory factor (MIF) can bind PINK1 and destabilize the PINK1–Parkin complex in epithelial cells, dampening mitophagy, escalating mitochondrial damage and oxidative burden, and ultimately reinforcing EMT and metastatic traits [179]. Beyond MIF–PINK1 crosstalk, IL‐33 signaling through suppression of tumorigenicity 2 (ST2)—its cognate receptor—emerges as a coordinated CAM program that couples mTOR‐driven metabolic rewiring with reduced mitochondrial turnover and an M2‐like polarization state, while an IL‐33–miRNA branch reinforces invasion and liver metastasis, together outlining a unified IL‐33‐driven mechanism linking macrophage mitochondrial control to dissemination [180, 181]. In sum, stromal mitophagy programs do not merely parallel tumor‐cell turnover; they operationalize intercellular control loops in which vesicle cargo and cytokine circuits re‐tune mitochondrial quality thresholds and metabolic bias across the stromal–tumor interface. CAF‐ and CAM‐centered pathways can, depending on the niche, either deliver mitochondria‐supportive cues or curtail mitophagy/lysosome‐dependent mitochondrial turnover, consequently tilting the balance between EMT‐leaning dissemination and restraint. These extrinsic control loops ultimately require molecular “writers” and “erasers” that specify mitochondrial substrate fate and signaling topology; accordingly, Section 4 dissects how ubiquitin E3 ligases and DUBs encode this regulatory layer at the mitochondria–EMT interface.

## 4 The Ubiquitin–Mitochondria Axis: Linking Organelle Quality Control to Metastatic Spread

The ubiquitin system supplies a versatile and context‐determined regulatory code that allows cells to dynamically recalibrate protein fate, signaling outputs, and organelle homeostasis. Through a hierarchical enzymatic cascade comprising ubiquitin activation (E1), conjugation (E2), and ligation (E3), offset by deubiquitinases (DUBs), ubiquitin marks take on signaling roles that go well beyond proteasomal turnover. Distinct patterns of ubiquitin chain topology—from mono‐ubiquitination and multi‐mono‐ubiquitination up to higher‐order polyubiquitin chains—encode regulatory information that partitions substrates toward degradation, signaling, trafficking, or metabolic adaptation [182]. Classical K48‐ and K11‐linked ubiquitination chiefly channels substrates to proteasome‐mediated degradation, whereas K63‐ and M1‐linked Ub chains are best known for non‐proteolytic signaling scaffolds, notably those that nucleate assembly of autophagy and inflammatory complexes [182]. Atypical polyUb architectures such as K6, K27, and K29 are now appreciated as context‐sensitive integrators of stress and proteome‐perturbing cues. Notably, ubiquitin conjugates bearing K6 or K27 linkages are used as signals for Parkin‐dependent mitophagy and broader MQC pathways, while K29 linkages have been linked to proteotoxic stress homeostasis and to the ubiquitin‐fusion degradation cascade, a dedicated proteasomal route for Ub‐appended substrates [183].

Appraised through the lens of mitochondrial functional plasticity, ubiquitin‐coded cues couple epithelial‐mesenchymal signaling circuitry to organelle state by coordinating metabolic resilience, morphodynamic remodeling, redox homeostasis, and mitophagy. These co‐regulated processes govern the reversibility of transitions between cohesive and motile cell states and the mechanistic logic of invasion introduced earlier. Under metastatic pressure, this integration must behave as an information‐processing pipeline: TME‐derived constraints are perceived, ubiquitin codes are encoded and decoded and EMT circuitry is reprogrammed in step with MQC outputs. Building on this premise, we parse the linkage between mitochondrial regulation and EMT governance into three ubiquitin‐directed modules across Sections 4.1–4.3: (i) modulation of EMT‐TF‐centered regulatory networks by integrating ubiquitin–proteasome system (UPS)‐dependent turnover with non‐proteolytic signaling to license transcriptional competence, (ii) retuning of OXPHOS capacity and fission–fusion homeostasis, (iii) refined control of receptor‐mediated and ubiquitin‐coupled mitophagy programs. In essence, this framework delineates how microenvironmental pressures elicit paired outputs—EMT program rewiring and mitochondrial maintenance—so that reversible state switching proceeds alongside the stress‐tolerance capacity required for metastatic fitness.

### 4.1 Context‐Sensitive Ubiquitin‐Dependent Regulation of EMT/MET Transcription‐Factor Stability, Functional Deployment, and Invasion Programs

#### 4.1.1 Context‐Tuned UPS Control of EMT/MET TF Half‐Life Window

Building on earlier sections that highlight transcription factor networks and chromatin landscape as organizers of epithelial–mesenchymal programs, we posit that transitions along the EMT–MET axis require more than shifts in gene‐expression output. They are also driven by post‐translational mechanisms that control regulator lifetime and complex assembly, providing the kinetic basis for reversible, bidirectional state changes. As such, a central question is how cells make phenotypic switching circuitry self‐resetting, allowing initiation of dispersal‐competent programs and eventual reacquisition of epithelial identity during re‐epithelialization [2]. One solution is to tune the half‐life of state‐transition effectors: the UPS can serve as a molecular timekeeper, delimiting the activity window of EMT‐associated drivers and their cofactors, and targeting them for removal to permit epithelial repolarization [184, 185]. Mechanistically, extracellular cues set this timing gate by modulating degron accessibility on plasticity‐linked transcription regulators, adjusting the balance between E3‐driven degradative ubiquitination and DUB‐mediated deubiquitination and specifying the point at which these factors become committed to proteasomal turnover.

At the protein‐quantity layer, Ub‐chain configuration specifies the stringency of 26S proteasome targeting: K48 poly‐Ub chains provide the dominant disposal signal, whereas K11‐linkages—often in mixed or branched formats—can further amplify proteasome docking and processing efficiency [186]. As an exemplar of this E3–DUB temporal coordination scheme, the SKP1–CUL1–RBX1–F‐box (SCF) complex configures E3 catalytic activity within the CUL1–RBX1 core and delegates substrate specificity to F‐box only protein 11 (FBXO11), thereupon committing SNAI1 to ubiquitin tagging and 26S‐mediated proteolysis. In contrast, Snail‐stabilizing DUBs—including members of the ubiquitin‐specific protease family (USPs) such as USP17 and USP27X—can reverse these degron‐activating modifications and sustain the nuclear‐accessible SNAI1 fraction [185]. During substrate–enzyme complex formation, kinase–DUB crosstalk can impose a selection checkpoint that potentiates DUB engagement toward EMT‐TFs. In TNBC, USP29—once phosphorylated and activated by cyclin‐dependent kinase 1 (CDK1)—strips ubiquitin chains from TWIST1, restraining proteasome‐directed turnover while sustaining EMT‐associated features, together with elevated motility and invasiveness in vitro [187]. On the MET side, an equivalent protein‐level gatekeeper is imposed by mouse double minute 2 homolog (MDM2), which nominates OVO‐like zinc finger 2 (OVOL2), an epithelial fate‐stabilizing TF, for ubiquitination and degradation, whereas p53 inhibits MDM2 to preserve OVOL2 and extend OVOL2‐dependent gene‐expression output [188].

With these substrate‐specific E3/DUB circuits organized to run a proteolytic timer, this abundance decision point is continually re‐timed under physiological microenvironmental constraints through three leverage points: E3‐ligase availability, degron priming, and proteasome‐delivery efficiency [189]. Recent in vivo studies illustrate that extracellular signals tune EMT/MET switching kinetics by gating UPS‐mediated turnover of core EMT‐TFs. In colorectal carcinoma, mitogen‐ and stress‐activated protein kinase 1 (MSK1) phosphorylates SNAI1 to license USP5 association, after which it deubiquitinates and stabilizes this pro‐dissemination driver, promoting motility/invasiveness and pulmonary metastatic burden after intravenous inoculation [190]. In lung cancer models, ubiquitin C‐terminal hydrolase L1 (UCHL1) cleaves K11‐ and K63‐linked ubiquitin chains on TWIST1; loss of this DUB diminishes TWIST1 protein abundance in an MG132‐sensitive manner, indicating that this enzyme normally buffers the substrate against 26S proteasome‐dependent degradation. This pattern is aligned with mixed or remodeled chain architecture (e.g., K11/K63 heterotypic chains), even though K63 linkages alone are typically regarded as proteasome‐independent. Functionally, UCHL1 knockdown suppresses migration/invasion and limits metastatic progression in vivo, with concordant effects in two models—reduced tumor burden after tail‐vein injection in BALB/c‐nude mice and constrained extravasation in zebrafish xenografts [191]. Beyond kinase–DUB licensing logic, canonical niche stresses can reshape the same UPS gatekeeper by rebalancing E3‐ligase availability and phosphodegron accessibility—e.g., hypoxia downregulates F‐box and leucine‐rich repeat protein 14 (FBXL14) (an SCF‐type E3 substrate receptor) to prolong the lifetime of SNAI1 via post‐translational stabilization without raising its transcript output; conversely, energetic stress (e.g., glucose deprivation) activates AMPK to phosphorylate this factor at Ser11, in turn priming its recognition by SCF^FBXO11 for ubiquitin attachment and expedited UPS‐linked clearance. In parallel, ECM mechanics stimulates Hippo signaling to route YAP/TAZ to the SCF E3 ligase via β‐transducin repeat‐containing protein (β‐TrCP), tuning their degradation and adding a separable control knob that widens or narrows the dwell time of pro‐migratory wiring [192, 193, 194]. Together, oxygen, metabolic, and biomechanical constraints can tune proteasome‐linked clearance kinetics of key state‐switching regulators, translating fluctuating niche inputs into adjustable protein half‐life (dwell‐time) windows that bias state persistence vs. termination.

#### 4.1.2 Non‐Degradative Ubiquitin “Deployment Codes” That Tune EMT/MET TF Nuclear Access and Transcriptional Potency

Beyond proteasome‐linked half‐life control, ubiquitination can function as a non‐degradative regulatory code that governs EMT/MET regulators through nuclear access, chromatin residence, and cofactor assembly. Whereas Section 4.1.1 highlighted degradative tagging as an “abundance timer,” this subsection focuses on how linkage architectures—often K63‐linked or mixed/mono‐ubiquitin configurations—reconfigure interaction surfaces and trafficking routes to modulate transcriptional efficacy without making protein abundance the primary driver. This layer is inherently context‐dependent: architecture‐encoded constraints (cell density, junction integrity, matrix mechanics) and microenvironmental pressures (e.g., inflammatory/immune cues) delimit when E3/DUB activities can tune these tags, while oncogenic rewiring driven by KRAS proto‐oncogene, can bias the same logic toward nuclear signaling states that favor invasion and metastatic fitness [184, 195, 196, 197, 198]. Therefore, the following two modules parse this non‐degradative ubiquitin logic into (i) Hippo/YAP–TEAD control of chromatin‐anchored transcriptional competence (Module I) and (ii) Wnt/β‐catenin nuclear‐engagement tuning via writer–eraser editing (e.g., RNF8 and USP13) (Module II), as summarized in Table 4.

**TABLE 4:** Architecture‐gated, non‐degradative ubiquitin tuning of nuclear signaling hubs linked to EMT plasticity.

Axis	Module I: Hippo/YAP–TEAD	Module II: Wnt/β‐catenin	Reference
Architecture gate	Tissue organization (contact/polarity/mechanics) sets YAP/TAZ nuclear permissiveness.	Junctional sequestration sets the signaling‐available β‐catenin fraction.	[199]
Representative ubiquitin logic	K63 writing/erasing tunes YAP–TEAD complex assembly and chromatin persistence.	K63 writing/erasing tunes β‐catenin nuclear engagement and TCF4 partnering.	[204, 205]
Writer / Eraser exemplars	Writer: TRAF6 → YAP1 (K63). Eraser: OTUD6A → TEAD4.	Writer: RNF8 → β‐catenin (K63). Eraser: USP13 → β‐catenin (K63, K508).	[200, 201, 203, 204]
Direct EMT‐TF coupling	YAP/TEAD cooperates with ZEB1 to activate pro‐motility targets (e.g., ITGA3).	β‐catenin/TCF4 directly induces ZEB1 transcription.	[201, 202, 205]
Functional outcome	Context‐tuned Hippo nuclear output supports invasion‐linked transcription and immune‐evasive programs.	Context‐tuned Wnt nuclear output crosses a ZEB1‐induction threshold that sustains EMT plasticity and invasion.	[202, 205]

Hippo/YAP–TEA domain transcription factor (TEAD) offers a mechanically gated transcriptional circuit in which junctional organization, polarity, and load set the threshold for YAP/TAZ nuclear entry and TEAD‐driven output. In tumors, Hippo deregulation sustains this nuclear program, while non‐degradative ubiquitin editing tunes complex assembly and persistence on chromatin rather than enforcing turnover [199]. Under inflammatory signaling, TNF receptor–associated factor 6 (TRAF6) serves as a K63 “writer” on Yes‐associated protein 1 (YAP1), enhancing its stability and promoting formation of a transcription factor CP2 (TFCP2)‐coupled complex, with programmed death‐ligand 1 (PD‐L1) as a functional response metric [200]. At the TEAD control point, OTU deubiquitinase 6A (OTUD6A) edits TEAD4‐linked ubiquitin without markedly changing TEAD4 abundance, strengthening YAP cofactor docking and increasing occupancy at target loci [201]. This chromatin‐anchored platform can then be co‐opted by ZEB1 to induce integrin subunit alpha 3 (ITGA3), linking Hippo nuclear output to adhesion and motility programs that support dissemination [202]. Overall, TRAF6‐dependent K63 writing and OTUD6A‐mediated editing define Module I as a tunable gain control that couples inflammatory tone to YAP/TEAD transcriptional potency and downstream EMT‐linked outputs.

A logically parallel module (Module II) is Wnt/β‐catenin, in which junctional architecture sequesters β‐catenin in cadherin‐based adhesive complexes and limits the fraction available for nuclear signaling. Beyond destruction‐complex–driven proteasomal turnover, non‐degradative ubiquitin editing can directly tune this signaling pool: in colon cancer, ring finger protein 8 (RNF8) installs K63‐linked polyubiquitin on β‐catenin to facilitate nuclear accumulation and strengthen Wnt target‐gene output [203]. Conversely, in KRAS‐mutant non‐small cell lung cancer, USP13—upregulated downstream of KRAS via Ras‐responsive element‐binding protein 1 (RREB1)—removes K63‐linked polyubiquitin from β‐catenin (reported at K508), enhancing β‐catenin engagement with transcription factor 7‐like 2 (TCF4) and metastatic phenotypes [204]. Crucially, β‐catenin/TCF4 can directly activate ZEB1 transcription, providing a mechanistic bridge from ubiquitin‐tuned nuclear β‐catenin partnering to core EMT circuitry and plasticity [205]. Taken together, Module II frames writer–eraser control as an efficiency dial that determines whether junction‐released β‐catenin is converted into a ZEB1‐priming transcriptional state that supports invasion, setting the stage for Section 4.2 on ubiquitin‐coded reprogramming of mitochondrial function during EMT.

### 4.2 Post‐Translational Reprogramming of Mitochondrial Function and EMT via Ubiquitin Code

Recent evidence underscores that tumor cells can reprogram both metabolism and morphology through substrate‐selective ubiquitin writing and erasing by specific E3 ligases and DUBs, without necessarily engaging overt mitophagy [189]. By tuning protein turnover and functional positioning, ubiquitin‐code‐guided cues remodel mitochondrial structure and bioenergetics—both by targeting metabolic enzymes mobilized throughout glycolysis, TCA cycle, and OXPHOS and by targeting morphogenetic effectors that control fission, fusion, and cristae maintenance [154]. The ensuing shifts in ATP production, redox balance and organelle dynamics lay the metabolic and structural underpinning for epithelial–mesenchymal phenotypic switching, fueling metastatic dissemination and later‐stage colonization.

**FIGURE 6:**
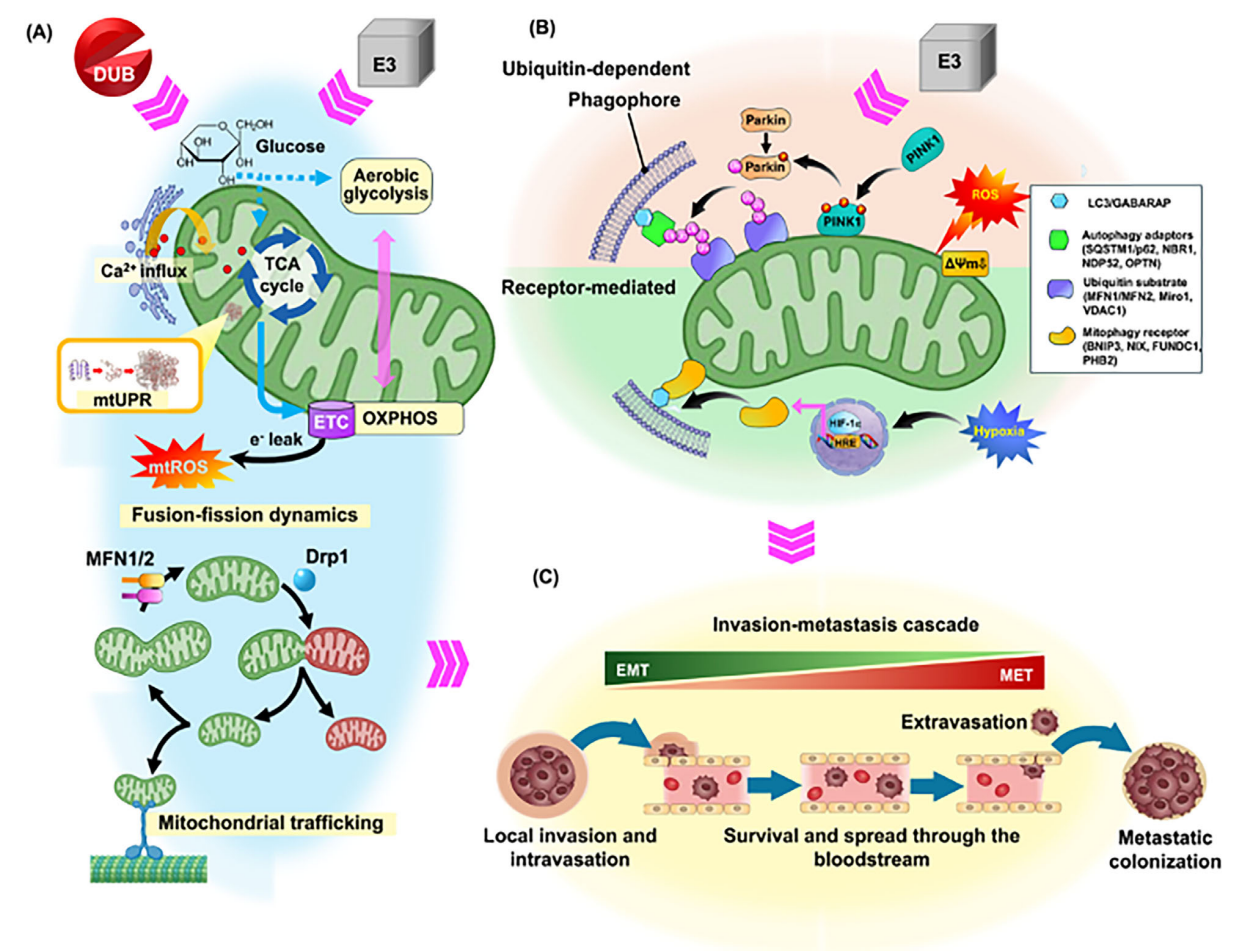
Crosstalk between ubiquitination pathways, mitochondrial homeostasis, mitophagy, and epithelial–mesenchymal plasticity in cancer metastasis. (A) The UPS, composed of E3 ubiquitin ligases and DUBs, finely tunes mitochondrial bioenergetics and structural dynamics, including metabolic flux between glycolysis and OXPHOS, mitochondrial fusion–fission balance mediated by Drp1 and MFN1/2, mtUPR, mtROS production, and Ca2+ influx. This multifaceted regulation reshapes mitochondrial trafficking and bioenergetic states, directly affecting EMT and metastatic potential. Detailed mechanisms and functional outcomes involving specific ubiquitin‐modifying enzymes (E3 ligases and DUBs) are systematically summarized and presented in Table 5. (B) Selective mitophagy pathways, encompassing ubiquitin‐dependent (i.e., PINK1–Parkin axis) and ubiquitin‐independent (receptor‐mediated) routes (via BNIP3, NIX/BNIP3L, FUNDC1, and PHB2), orchestrate mitochondrial clearance to maintain cellular homeostasis. In ubiquitin‐dependent mitophagy, loss of ΔΨm stabilizes PINK1 on the mitochondrial outer membrane, recruiting and activating Parkin to ubiquitinate mitochondrial substrates (e.g., MFN1/2, VDAC1, and Miro1). This signal recruits autophagic adaptors (e.g., SQSTM1/p62, NBR1, NDP52, and OPTN) to initiate autophagosome formation. Conversely, receptor‐mediated mitophagy occurs independently of ubiquitination: under hypoxia, HIF‐1α transcriptionally activates BNIP3 and NIX, exposing their LIR for direct LC3 binding, while FUNDC1 is activated by dephosphorylation at Ser13 (by PGAM5) and phosphorylation at Ser17 (by ULK1). Mechanistic details comparing ubiquitin‐dependent and receptor‐mediated mitophagy pathways are summarized in Table 5.

To keep this section mechanistically coherent, we organize ubiquitin‐based mitochondrial rewiring into three functional tiers: (i) metabolic and signaling‐node control, (ii) remodeling of mitochondrial dynamics and stress responses (mtROS/mtUPR), and (iii) higher‐order organization (positioning, cristae architecture, and ER–mitochondria contact‐site signaling) that sculpts motility‐relevant cell states [30, 206]. As schematically illustrated in Figure 6A and compiled in Table 5, E3 ligases together with DUBs coordinate mitochondrial energy metabolism, structural transitions (fission–fusion via Drp1 and MFN1/2), mtUPR activation, mtROS homeostasis, and Ca^2^^+^ signaling, which shape EMT‐related features and metastatic fitness. A growing repertoire of E3 ligases senses cues such as hypoxia, ROS burden, and matrix rigidity and recalibrates their substrate portfolio accordingly, driving microenvironment‐specific transitions along the EMT–MET axis [207].

**TABLE 5:** Ubiquitin editing of mitochondria: Enzymatic nodes linking metabolism to EMT and metastasis.

Category	Impact	Enzyme	Intrinsic factors	Extrinsic cues	Substrate	Action	EMT/Migration	Reference
Bioenergetics	Metabolic shift	Skp2 (SCF E3)	Skp2 level	Hypoxia, ROS burden, matrix rigidity	AKT	Adds K63 chains → AKT activation	cancer stem cell (CSC) expansion, EMT, invasion	[208]
	Metabolic shift	TRIM28 (TRIM‐family E3)	TRIM28 level	N.D.	FBP1	Polyubiquitinates FBP1 → depletion	↑ Invasiveness (HCC)	[209]
	Sustains TCA & OXPHOS	USP13 (DUB)	USP13 level	N.D.	ACLY, OGDH	DeUb (K48) ACLY/OGDH → stabilization	↑ USP13 →↑ EMT & invasion; ↓ USP13 →↓ metastasis	[210]
Fusion‐fission dynamics	Excess fission,↑ ROS	MARCH5 (E3)	MARCH5 level	ROS	Drp1	Promotes Drp1‐mediated fission → ↑ ROS	EMT & metastasis (breast cancer)	[211]
	Excess fission	USP9X (DUB)	USP9X level	N.D.	Drp1	Maintains dynamics; loss → Drp1Ser616 phosphorylation loss → Drp1Ser616 phosphorylation	USP9X loss → ↑ migration & invasion	[260]
Mitochondrial stress	Glutathione loss,↑ mtUPR	RNF148 (E3)	RNF148 level	Redox imbalance	CHAC2	Targets CHAC2 to proteasome → GSH↓, mtUPR	↑ GIcancer motility	[212]
Mitochondrial trafficking	Immobilization	CHIP (E3)	CHIP activity	N.D.	Syntaphilin	K63Ub, nondegradative	↓ Chemotaxis; loss of K63‐Ub → ↑ invasion	[213]
Mitochondrial integrity	Cristae loss, ΔΨm collapse	UPS (E3 N.R.)	Proteasome activity	Low pH	MIC60	Proteasomal turnover of MIC60 (MICOS disruption)	Energy deficit; invasive adaptation	[214]
Mitochondrial calcium uptake	↓ ERmito Ca^2^^+^ transfer	FBXL2 (SCF E3)/ BAP1 (DUB)	PTEN loss, BAP1 activity	N.D.	IP3R3	FBXL2 Ub → IP3R3 depletion; BAP1 deUb rescues	Apoptosis resistance, migration	[38]

Metabolic and signaling‐node control: For example, as an SCF‐associated E3 ligase component, S‐phase kinase‐associated protein 2 (Skp2) decorates AKT with K63‐linked chains, consequently boosting AKT phosphorylation and signaling output. This AKT activation promotes expansion of cancer stem cell‐like populations and—given AKT's place in EMT and cytoskeletal remodeling—provides a direct route to invasive traits [208]. Taking this logic beyond kinase signaling, ubiquitin circuitry can also recast invasion by reshaping metabolic flux and mitochondrial dynamics. In HCC, tripartite motif (TRIM) family member TRIM28 enforces depletion of fructose‐1,6‐bisphosphatase, rerouting HCC metabolism toward glycolysis and heightening invasive phenotypes [209]. Within this ubiquitin–mitochondria regulatory axis, USP13 stabilizes ATP‐citrate lyase and oxoglutarate dehydrogenase by removing K48‐linked ubiquitin chains, sustaining acetyl‐CoA supply, TCA‐cycle flux, oxidative phosphorylation, and redox/energy homeostasis. Consistently, USP13 overexpression increases EMT‐marker expression and invasiveness, whereas USP13 depletion reduces oxygen consumption and diminishes metastatic capacity [210].

Dynamics/stress responses (fission–fusion, mtROS, and mtUPR): The outer‐membrane (OMM) ligase membrane‐associated RING‐CH protein 5 (MARCH5) elicits Drp1‐mediated mitochondrial fission and ROS accrual, which has been identified as a driver of EMT and metastatic progression in breast cancer models [211]. Along the same stress‐response axis, RNF148 targets ChaC glutathione‐specific gamma‐glutamylcyclotransferase 2 (CHAC2) for proteasomal degradation, depleting glutathione and triggering a redox‐imbalanced mitochondrial unfolded protein response (mtUPR) that disrupts mitochondrial integrity and accelerates gastrointestinal cancer cell motility and peritoneal dissemination [212].

Higher‐order mitochondrial organization (positioning, cristae, and ER‐mitochondria contact sites): Aside from metabolic regulation and fission–fusion dynamics, ubiquitination can also tune tumor‐cell motility by redirecting higher‐order mitochondrial positioning. C‐terminus of Hsc70‐interacting protein (CHIP) installs K63‐linked polyUb chains on syntaphilin, anchoring mitochondria to cortical microtubules and limiting their trafficking to the leading edge, as a result suppressing chemotaxis and experimental metastasis; loss of this non‐degradative modification removes the restraint and enhances invasion [213]. At an even deeper architectural tier, ubiquitin‐dependent proteasomal turnover can remodel inner‐membrane scaffolds that sustain cristae organization. In acidic TMEs, rapid proteolysis of mitochondrial contact site and cristae organizing system subunit 60 (MIC60; mitofilin) disrupts the mitochondrial contact site and cristae organizing system (MICOS) complex, collapsing cristae structure and mitochondrial membrane potential with a concomitant loss of ATP output [214]. At ER–mitochondria contact sites, loss of phosphatase and tensin homolog (PTEN) deleted on chromosome 10 unleashes the F‐box substrate receptor F‐box and leucine‐rich repeat protein 2 (FBXL2) to deplete the Ca^2^^+^ channel inositol 1,4,5‐trisphosphate receptor type 3 (IP3R3) via ubiquitin‐dependent turnover, in turn blunting ER‐to‐mitochondria Ca^2^^+^ flux and favoring an antiapoptotic, motile state that BRCA1‐associated protein 1 (BAP1), a DUB, can reverse [38]. In contrast to the EMT‐TF–timing F‐box node discussed in Section 4.1, this MAM‐localized FBXL2 axis channels the same SCF logic into Ca^2^^+^‐coupled survival and motility wiring.

These E3 ligase– and DUB‐regulated mitochondrial nodes, together with their downstream effects on EMT/MET switching and metastatic behaviors and contextual microenvironmental signals, are compiled in Table 5. A “threshold” model posits that ubiquitin‐dependent remodeling first acts as a reversible, pre‐mitophagy adaptive layer—buffering or rewiring metabolism, dynamics, cristae integrity, and contact‐site Ca^2^^+^/stress signaling—whereas only once cumulative stress surpasses a context‐defined limit are mitochondria routed into mitophagy [30, 206, 215]. As schematized in Figure 6 (see panel C), ubiquitin editing can be viewed as a context‐tunable governor that often acts in a reversible, pre‐mitophagy mode to preserve mitochondrial homeostasis and metabolic flexibility, but can switch—once cumulative stress burden breaches a context‐defined threshold—to engage mitophagy programs that refurbish organelle quality along the invasion‐to‐colonization continuum. This threshold‐to‐mitophagy handoff sets the stage for Section 4.3, which dissects how distinct microenvironmental pressures bias mitophagy entry routes and effector deployment, shaping EMT–MET transitions. See Box 2 for a speculative view of how histone‐targeting DUBs might couple nuclear chromatin regulation to mtUPR gene expression. Overall, Section 4.2 frames ubiquitin architectures as a context‐sensitive ‘gain control’ for EMT/MET transcriptional hubs, tuning nuclear residency and cofactor assembly in parallel with metabolic stress and cytoskeletal demand. This logic sets up Section 4.3, where we map mitochondria‐localized E3/DUB circuits that gate MQC/mitophagy flux in space and time.

### 4.3 Ubiquitin‐Dependent Versus Receptor‐Mediated Mitophagy: A Context‐Tuned Rheostat for Tumor Invasion

#### 4.3.1 Pathway Overview—Two Entry Routes, One Lysosomal Endpoint

Mammalian cells uphold mitochondrial quality via two complementary yet mechanistically distinct mitophagy pathways that converge on the lysosomal degradation of damaged organelles. In the ubiquitin‐dependent route, loss of ΔΨm blocks import and cleavage of PINK1 by PARL, causing full‐length PINK1 to build up on the OMM, where it phosphorylates ubiquitin at Ser65 and recruits the RBR‐type E3 ligase Parkin [119]. Parkin becomes further activated through PINK1‐driven phosphorylation of its ubiquitin‐like (UBL) domain, thereafter translocating to membrane potential‐dissipated mitochondria and catalyzing the installation of mixed K6‐, K11‐, K48‐, and K63‐linked ubiquitin chains on OMM targets including MFN1/2, voltage‐dependent anion channel 1 (VDAC1), and mitochondrial Rho GTPase 1 (Miro1) [216]. These ubiquitin modifications are recognized and read out by adaptor proteins bearing both ubiquitin‐associated domain (UBA) and LC3‐interacting region (LIR) motifs—representative examples include sequestosome 1 (SQSTM1, also known as p62), next to BRCA1 gene 1 protein (NBR1), nuclear dot protein 52 kDa (NDP52), and optineurin (OPTN). These adaptors mediate docking, recruiting ubiquitin‐decorated mitochondria to LC3‐ or gamma‐aminobutyric acid receptor‐associated protein (GABARAP)‐enriched phagophores, thus driving autophagosome biogenesis and maturation [217].

A distinct selection mechanism operates in receptor‐mediated (often termed Parkin‐independent mitophagy pathway), which depends on dedicated OMM proteins—BNIP3, NIX/BNIP3L, and FUNDC1—that engage LC3 through intrinsic LIR motifs. Under hypoxia, HIF‐1α transcriptionally upregulates BNIP3 and NIX, whose glycine–zipper‐driven dimerization then exposes LIR motifs, promoting LC3 binding [218]. Meanwhile, FUNDC1 remains dormant under basal conditions via casein kinase 2‐ and Src‐mediated phosphorylation at Ser13 and Tyr18; following ΔΨm collapse or hypoxic insult, PGAM5 removes the Ser13 phosphate and ULK1 adds a phosphate at Ser17, converting FUNDC1 into an active LC3‐binding receptor that coordinates mitophagic engulfment [219]. As shown in Figure 6B, the mitophagy machinery—comprising the ubiquitin‐reliant PINK1–Parkin axis and the receptor‐driven BNIP3, NIX, FUNDC1, and PHB2 pathways—executes mitochondrial clearance to sustain cellular homeostasis, with comparative details summarized in Table 6. In combination, these paired “tag‐and‐bridge” vs. “direct‐receptor” entry routes enable mitophagy to operate as a graded, context‐tuned rheostat rather than an all‐or‐none switch—buffering mitochondrial stress while preserving the potential for organelle‐level attrition when damage and microenvironmental pressure escalate [17, 31].

**TABLE 6:** Comparative impact of ubiquitin‐dependent and receptor‐mediated mitophagy on EMT/MET plasticity and metastatic traits ^*^Selected mitophagy‐independent Parkin substrates are included to illustrate axis‐level context dependence (Section 4.3.3).

Route	Intrinsic regulators	Extrinsic cues	EMT/MET	Migration/Metastasis	Key mechanistic notes	Reference
Ubiquitin‐dependent	DUB threshold: USP30/USP15; facilitator USP8; TBK1→OPTN	ΔΨm loss; mitochondrial stress load	Flux gating biases EMT/MET via mitochondrial fitness	Rheostat for pro vs antimotility outputs	Commitment when Ub writing exceeds DUB brakes; TBK1 amplifies adaptor avidity	[227, 228]
	PINK1, Parkin, SQSTM1/p62	Mitochondrial depolarization, OXPHOS demand	Drives full EMT (↑ SNAI1, vimentin, Ncadherin)	Promotes lymphnode dissemination	p62 supports Parkin polyubiquitin signaling to sustain OXPHOS and EMT transcriptional output	[230]
	SFXN1 (blocks PINK1 docking/accumulation)	mtROS rise, TGFβ signaling	Favors EMT via ROSsensitized TGFβ pathway	Facilitates lung colonization; Parkin reactivation reverses	Mitophagy dampening → mtROS buildup → EMT trigger	[231]
	ABALON (lncRNA; accelerates PINK1/Parkin turnover)	Therapy pressure (5fluorouracil)	Enriches EMT hallmarks (CMS1 colorectal tumors)	Supports chemoresistance and aggressive traits	Fast turnover maintains mitochondrial fitness and advances 5FU resistance	[232]
	Parkin → catalase	Redox stress tone	Shifts redox balance (↑ intracellular ROS)	Limits migration (bladder cancer)	Catalase ubiquitination elevates ROS to restrain motility (mitophagyindependent)	[233, 234]
	Parkin → Kindlin2	Adhesion/motility programs	Attenuates β1integrin activation	Reduces lamellipodia dynamics and motility (breast cancer)	Proteasomal turnover of Kindlin2 dampens focaladhesion output (mitophagyindependent)	[233, 234]
	Parkin → HIF1α	Hypoxia	Maintains epithelial bias under hypoxia	Restrains hypoxiadriven migration (breast cancer)	Lys477 ubiquitination targets HIF1α for proteasomal degradation (mitophagyindependent)	[235]
	PARK2 loss/LOH or null variants	Gene dosage (tumor genomics)	Removes Parkinmediated restraint	Tumorpromoting consequences in vivo (ApcMin/+ intestinal adenoma)	Reduced PARK2 dosage ablates motility/tumorsuppressive outputs	[236, 237]
Receptor‐mediated	Contactsite calibration: MARCH5 vs USP19 (FUNDC1); Drp1 coupling	Hypoxia; nutrient limitation; ROS	Stressactivated receptors coordinate EMTadaptive remodeling	Aligns fissioncapture for survival/anoikis resistance	ER–mitochondria interfaces tune receptor availability and fragment capture	[211]
	BNIP3, FUNDC1	Cirsiliol binding to STAT3	Loss of receptor signaling collapses ΔΨm	Impairs scratchwound closure (reduced migration)	Dual suppression of receptor and Parkin axes → ΔΨm loss, ↑ ROS	[238]
	BNIP3 (loss)	Hypoxia	ROS accumulation drives EMT	Enhances metastatic spread	Failure to clear damaged mitochondria amplifies ROS and EMT	[138, 239, 240]
	NIX/BNIP3L	Oncogenic KRAS signaling	Supports metabolic flexibility (partial EMT)	Promotes tumor dissemination	Increased receptor mitophagy matches energy demand of motile cells	[138, 239, 240]
	FUNDC1	Hypoxia, metabolic stress	Confers anoikis resistance	Facilitates survival of detached cells	Active FUNDC1LC3 binding stabilizes detached mitochondrial fragments	[138, 239, 240]
	BNIP3/NIX, FUNDC1 (stressactivated)	Cisplatin cytotoxic stress	Induces G0like dormancy	Creates chemoresistant reservoir that seeds later metastasis	Sustained receptor clearance supports quiescent survival	[241]

#### 4.3.2 Decision Logic: DUB‐Thresholds, Contact‐Site Calibration, and Kinase Amplification

The decision to engage ubiquitin‐dependent vs. receptor‐mediated mitophagy hinges on a finely orchestrated crosstalk between cell‐intrinsic regulators and extracellular cues, with “commitment” emerging when pro‐mitophagy signals exceed pathway‐specific inhibitory brakes. Inside cells, the PINK1/Parkin pathway detects mitochondrial injury, but mitophagy proceeds only when ubiquitin signaling overcomes a DUB‐imposed threshold: USP30 and USP15 erase Parkin‐built chains, whereas USP8 deubiquitinates Parkin to sustain its activity [220, 221]. Receptor‐mediated mitophagy, by contrast, is more directly driven by microenvironmental stress (hypoxia, nutrient limitation, ROS) and is tuned at ER–mitochondria contact sites through opposing ubiquitin enzymes: MARCH5 promotes FUNDC1 turnover, whereas USP19 stabilizes FUNDC1 to sustain high‐affinity LC3 binding under hypoxia [222]. At a systems level, this contact‐site module also aligns mitochondrial fragmentation with receptor output: MARCH5–USP19 control over FUNDC1 is coupled to Drp1‐driven fission, ensuring that LC3‐competent receptors and engulfable mitochondrial segments are generated in register for efficient capture.

Molecularly, Parkin's wide‐ranging ubiquitination constructs branched K6/K63 chain architectures that are parsed by adaptors using UBA/ubiquitin‐binding in ABIN and NEMO (UBAN) domains and LIR motifs, fastening ubiquitin‐marked mitochondria to early phagophore membranes [183]. Beyond the PINK1/Parkin axis, the repertoire of mitochondrial ubiquitination is broadened by alternative E3 ligases—such as mitochondrial ubiquitin ligase 1 (MUL1), glycoprotein (Gp78), Ariadne RBR E3 ubiquitin protein ligase 1 (ARIH1), and SMAD ubiquitination regulatory factor 1 (Smurf1) [223, 224, 225, 226]—which introduce varied chain types and branch points. DUBs like USP34 and the facilitator USP8 prune these chains to calibrate receptor engagement, while TANK‐binding kinase 1 (TBK1) phosphorylates cargo adaptors such as OPTN to boost their ubiquitin and LC3‐binding affinities, potentiating mitophagic flux [227]. Recent research suggests that TBK1 adaptor modules tune the efficiency of mitophagy progression, supporting a view that pathway choice and strength emerge from multi‐node rheostats rather than a single checkpoint [228].

Importantly, receptor‐mediated mitophagy couples clearance signaling to mitochondrial dynamics: BNIP3/NIX dimers expose their LIR motifs at Drp1‐driven fission sites for LC3/GABARAP binding, whereas FUNDC1's LIR is switched on by stress‐regulated phosphorylation cycling. Both receptor classes then promote Drp1 recruitment via Fis1/mitochondrial fission factor (Mff) and dampen OPA1‐dependent fusion, focusing autophagosome formation on discrete mitochondrial fragments. [229]. Taken together, ubiquitin‐chain proofreading, phospho‐enhanced adaptor avidity, and ER–mitochondria interface calibration converge to define when damaged mitochondria are effectively handed off to LC3/GABARAP‐coated isolation membranes, steering clearance via Parkin‐based vs. receptor‐centered entry modes in a niche‐imposed stress state–dependent manner.

#### 4.3.3 Context‐Dependent Outputs of the PINK1–Parkin Axis—Bidirectional Coupling to EMT/MET Plasticity

In diverse tumor settings, the PINK1–Parkin ubiquitin cascade can steer epithelial–mesenchymal phenotypic switching. In intrahepatic cholangiocarcinoma, SQSTM1/p62 supports Parkin‐driven polyubiquitin assembly on damaged mitochondria, upholding OXPHOS and upshifting SNAI1, vimentin, and N‐cadherin to reinforce EMT‐like phenotypes and lymph‐node metastasis [230]. In bladder cancer, the same axis can be redirected toward epithelial identity: increased sideroflexin‐1 (SFXN1), a mitochondrial serine transporter, prevents PINK1 docking and dampens mitophagy, precipitating mtROS rise with consequent TGF‐β pathway activation that favors EMT and pulmonary seeding; in turn, restoring Parkin activity—genetically or pharmacologically—reinstates epithelial features and curbs metastatic spread [231]. A third layer of control is provided by the apoptotic BCL2L1‐antisense long non‐coding RNA (ABALON), which accelerates PINK1/Parkin turnover to maintain mitochondrial fitness, advance 5‐fluorouracil resistance, and enrich EMT hallmarks in consensus molecular subtype 1 (CMS1) colorectal tumors [232].

Apart from organelle‐directed clearance, Parkin can suppress invasive behavior by ubiquitinating non‐mitochondrial effectors that tune redox handling, adhesion signaling, and hypoxia responses (Table 5). In this mitophagy‐independent setting, catalase ubiquitination rebalances antioxidant buffering to raise signaling‐competent ROS and restrict bladder cancer cell movement, whereas degradative tagging of the focal‐adhesion regulator Kindlin‐2 weakens β1‐integrin activation and dampens lamellipodial dynamics in breast cancer cells [233, 234]. Extrapolating the same principle to oxygen‐sensing circuitry, Parkin designates HIF‐1α at Lys477 for proteasomal turnover, blunting hypoxia‐driven migratory behavior [235]. Conversely, focal copy‐number depletion or null variants at the Parkin RBR E3 ubiquitin protein ligase (PARK2) locus—reported in roughly one‐third of sporadic colorectal carcinomas and frequently in glioblastoma—ablate this restraint; echoing this idea, monoallelic PARK2 loss in Apc^Min/+ mice drives intestinal adenoma formation, revealing the tumor‐promoting downside of Parkin insufficiency [236, 237]. In mechanistically terms, the PINK1–Parkin module functions as a bifurcating ubiquitin editing program: one branch adjusts organelle quality through mitochondria‐directed turnover pathways, whereas the other delivers a subset of motility‐linked redox, adhesion, and hypoxia effectors to the proteasomal machinery. Dominance between these outputs is reweighted by the stress landscape and PARK2 allelic status, yielding phenotypes that resist a simple tumor‐suppressor vs. tumor‐promoter classification. Accordingly, whether Parkin checks or promotes metastatic traits—and to what extent—tracks with its target spectrum and PARK2 dosage.

#### 4.3.4 Receptor‐Mediated Mitophagy—Stress Sensing, Motility Control, and Therapy‐Driven Dormancy

Classical mitophagy receptors bearing bona fide LIR motifs—notably the OMM proteins BNIP3, NIX/BNIP3L and FUNDC1 (and, in specific Parkin‐primed settings, the IMM receptor prohibitin 2 (PHB2) exposed after OMM rupture)—provide a rapid, direct LC3/GABARAP‐docking conduit for MQC that can concomitantly sculpt motile phenotypes [119]. Selected invasion‐relevant cancer examples are highlighted in Table 6. In colorectal cancer cells, the flavonoid cirsiliol inhibits both receptor‐mediated (BNIP3/FUNDC1) and PINK1–Parkin mitophagy—via STAT3 engagement—leading to ΔΨm loss, ROS elevation, and slower wound‐healing closure, all of which are rescued by pharmacological reinstatement of mitophagic activity [238]. Across cancer contexts, genetic or expression‐level perturbations in receptor‐type mitophagy nodes repattern how tumor cells buffer mitochondrial stress triggered by hypoxia, oncogenic signaling, or ECM detachment. These shifts propagate to redox balance, metabolic flexibility, and anchorage survival: under hypoxia, BNIP3 loss allows ROS‐driven EMT programs; in KRAS‐driven settings, NIX/BNIP3L induction supports bioenergetic adaptability; and elevated FUNDC1 strengthens anoikis resistance—together tilting cells toward metastatic spread and colonization [138, 239, 240]. Independent of immediate effects on motility, therapy‐sustained receptor‐mediated clearance can also select for a dormant, drug‐tolerant state. For example, cisplatin‐treated head‐and‐neck squamous‐cell carcinoma cells enter a G_0_‐like, stemness‐high quiescence driven by mitophagy; upon drug withdrawal, they seed aggressive metastases—an effect mitigated by autophagy blockade [241]. This treatment‐entrained “mitophagy‐for‐survival” mode fits with broader evidence that mitophagy can be co‐opted to support cancer adaptation, therapy resistance, and relapse in a tumor‐ and stage‐dependent manner [10, 17]. Hence, mitochondrial receptors act as sensors of hypoxia, inflammatory cytokines and drug pressure, translating metabolic cues into either migratory capacity or survival state switching without relying on ubiquitin tags. As depicted in Figure 6C, the crosstalk between ubiquitination–deubiquitination and selective mitophagy routes dynamically regulates mitochondrial integrity and metabolic adaptability, governing cellular shifts along the EMT–MET spectrum throughout the invasion–metastasis cascade. Tumor cells co‐opt these mitochondrial control networks to drive local tissue invasion, intravasation, survival in circulation, extravasation, and final metastatic colonization. Altogether, mitochondria‐embedded E3/DUB modules impose spatial and temporal specificity on ubiquitin labeling, as a result controlling when damaged organelles are cleared versus retained to sustain motile bioenergetic output. This coupled ‘ubiquitin–mitophagy circuit’ motivates the translational opportunities and constraints discussed in Section 5.

## 5 Translational and Therapeutic Opportunities Along the Ubiquitin–Mitophagy Circuitry: Challenges and Future Directions in Metastasis

Across Sections 2, 3, 4, ubiquitin writing/erasing and mitophagy flux emerge as a coupled control circuit that shapes mitochondrial stress outputs and, in turn, metastatic state transitions in a context‐dependent manner. Section 5 translates this circuitry into therapeutic logic by mapping actionable intervention nodes (where the circuit can be tuned) and outlining translational bottlenecks (delivery/safety and flux‐resolved biomarkers) that determine whether anti‐metastatic benefit can be tested rigorously in vivo and in patients. With this circuit view, Section 5.1 organizes current strategies as entry points that (i) impose pro‐mitophagy ubiquitin signals on mitochondria, (ii) prevent their erasure, or (iii) lower the threshold and raise throughput for mitochondrial clearance. In sum, therapeutic leverage on the ubiquitin–mitophagy axis hinges on aligning target‐node selectivity with organelle‐level delivery while reading out true mitophagy flux rather than static markers. These constraints naturally lead to the outstanding mechanistic and experimental questions outlined in Section 5.2.

### 5.1 Therapeutic Strategies Targeting the Ubiquitin–Mitophagy Axis

Therapeutic strategies that engage the ubiquitin–mitophagy machinery converge on a shared objective: to shorten the lifetime of dysfunctional mitochondria that otherwise sustain signaling‐competent stress outputs. A first entry point is to “write” a mitophagy‐competent ubiquitin coat on the OMM in a Parkin‐like manner. Autophagy‐targeting chimeras have been reported to promote mitophagy by chemically tethering mitochondria to the autophagy machinery, and in some designs are accompanied by enhanced K63‐linked ubiquitination on the mitochondrial outer membrane, thereby facilitating ubiquitin‐dependent recruitment of autophagy adaptors and mitochondrial turnover [242]. Mechanistically, this reframes proteolysis‐targeting chimera (PROTAC)‐like design as a programmable ubiquitin‐writing tool that can reset mitochondrial stress signaling upstream of pro‐migratory pathways in contexts where clearance failure is the dominant liability [231].

A second entry point is to preserve or amplify pro‐mitophagy ubiquitin signals by downregulating mitochondria‐localized DUB activity. USP30 counterbalances Parkin by removing ubiquitin from mitochondrial substrates; accordingly, USP30 suppression increases mitochondrial ubiquitin burden and can restore mitophagy in impaired settings [243]. In parallel, immune‐focused evidence indicates that USP30 blockade can rejuvenate mitochondrial health in tumor‐infiltrating T cells and enhance anti‐tumor function [244]. Together, these data motivate a compartment‐aware translational framing: mitochondrial ubiquitin editing may be leveraged in tumor and/or immune cells, but the optimal compartment and intervention timing are expected to hinge on metastatic stage and microenvironmental constraints.

A third entry point is to lower the activation threshold and/or increase downstream clearance throughput. Small molecules that stabilize PINK1 on damaged mitochondria can facilitate Parkin recruitment, ubiquitin tagging, and mitophagy initiation [245]. Conversely, agents such as urolithin A can enhance TFEB‐linked autophagy–lysosomal programs, increasing clearance capacity under high damage load [245]. Representative emerging pharmacologic modulators of ubiquitin–mitophagy signaling (including mitophagy inducers, USP30 inhibitors, and PINK1‐stabilizing/activating strategies) are summarized in Table S1, with molecular structures, primary targets, and proposed regulatory mechanisms. Viewed together, these modalities “tune” a single circuit—ubiquitin tagging, ubiquitin editing, and lysosomal throughput—supporting the idea that anti‐metastatic efficacy will be maximized by matching the entry point to the dominant failure mode in a given tumor context. However, converting these entry insights into viable therapies is not straightforward. Translation is constrained by context selection (who benefits and when), delivery/safety (where the drug accumulates), and flux‐aware readouts that demonstrate pathway‐specific engagement longitudinally.

### 5.2 Outstanding Questions and Future Research Directions

Outstanding Question 1: Which tumor contexts interpret ubiquitin–mitophagy circuit engagement as a brake on dissemination versus a support for stress tolerance and colonization? A central priority is to operationalize “context selection” rather than assuming uniform benefit from boosting or suppressing mitophagy, because the ubiquitin–mitophagy axis can buffer metastatic stress yet, in other settings, sustain survival advantages. Accordingly, the clinically actionable question is not simply whether mitophagy changes, but which tumor contexts render circuit engagement anti‐dissemination versus pro‐tolerance, motivating systematic cross‐context testing that explicitly separates dissemination‐stage vulnerabilities from colonization‐stage requirements rather than extrapolating from single‐model effects.

Outstanding Question 2: What flux‐resolved PK/PD readouts can distinguish “pathway engaged” from “clearance blocked” in clinically tractable samples? Measurement remains a limiting bottleneck because mitophagy is a flux process, yet clinical inference is often drawn from single time‐point markers that cannot distinguish increased initiation from impaired downstream clearance. Although mt‐Keima and mito‐QC enable in vivo flux tracking preclinically, they are not clinically deployable [119]. Thus, a key priority is to develop repeatedly sampleable PK/PD‐style assays that report pathway‐proximal engagement, including ubiquitin‐on‐mitochondria events together with lysosomal delivery/throughput, enabling interpretation that separates “pathway engaged” from “clearance blocked” [119].

Outstanding Question 3: Which delivery‐enabled strategies can achieve tumor‐enriched exposure and functional circuit rewiring, and how should they be validated efficiently? Translation is bounded by delivery and tolerability because mitochondria are indispensable in high‐energy tissues, and broad mitochondrial perturbation can be dose‐limited by systemic toxicity [246, 247]. PROTAC‐like modalities also face size‐driven penalties in permeability, oral bioavailability, and intratumoral penetration [248], motivating pro‐PROTAC designs triggered by tumor‐associated cues and delivery‐calibrated formulations, including nano‐enabled approaches that raise effective tumor exposure by facilitating cellular entry via endocytosis rather than passive diffusion [248, 249]. To de‐risk exposure–response and biomarker hypotheses while reducing overreliance on animal studies, validation should follow a staged pipeline that retains TME constraints yet standardizes pharmacokinetics/pharmacodynamics (PK/PD): Patient‐derived organoid (PDO) panels for longitudinal 3D testing [250], Patient‐derived explants (PDEs) for short‐window functional testing and biomarker discovery [251], and cuboid‐style live microdissected tumor fragments in valved multiwell microfluidic platforms for higher‐throughput, diffusion‐barrier–relevant pharmacotyping [252], reserving limited in vivo studies for late‐stage confirmation in site‐relevant and immunocompetent metastasis model. As summarized in Table 7, translating the ubiquitin–mitophagy circuit into anti‐metastatic therapy will hinge on context selection across dissemination versus colonization, tumor‐enriched delivery to expand the therapeutic window, and flux‐resolved PK/PD biomarkers that report longitudinal pathway engagement.

**TABLE 7:** Therapeutic strategies and translational challenges targeting the ubiquitin–mitophagy axis in metastasis.

Therapeutic Entry Point	Representative Modalities	Role in Metastasis & EMT	Translational Challenges	Future direction	Reference
Inhibition of OXPHOS	ETC inhibitor	Context‐dependent: inhibits early metastatic events, enhances late‐stage progression	Target specificity; systemic toxicity	Targeted delivery system	[246, 247]
Mitochondrial Ub writing	Mitophagy‐enhancing chimeras / PROTAC‐like tools		Large molecular size limits permeability & delivery.	pro‐PROTACs (tumor‐cue triggered) and nano‐enabled delivery.	[248, 249]
Block Ub erasing (DUB)	USP30 Inhibitors		Context selection: Determining if circuit engagement acts as a "brake" on dissemination or "support" for survival.	Timing interventions for tumor vs. immune cells based on metastatic stage	[243, 244]
Lower mitophagy activation threshold	PINK1 Stabilizers		Single time‐point markers are insufficient.	Requires flux biomarkers to development PK/PD assays and distinguish "pathway engaged" from "clearance blocked" longitudinally	[245]
Upregulates mitophagy	Mitophagy Inducers (e.g. Urolithin A)				[245]

## 6 Conclusion and Perspectives

Rather than a single steady mitochondrial pathway, metastasis derives from a dynamic system that continually resets energy metabolism, ROS signaling, mitochondrial fission–fusion, and targeted organelle clearance in response to intrinsic programs, extrinsic microenvironmental cues, and stage‐/site‐specific constraints along the invasion–metastasis cascade. Central to this adaptability is the UPS, which edits the lifespan and activity of metabolic enzymes, mitochondrial‐quality regulators, and EMT‐TFs via an ever‐expanding repertoire of canonical and non‐canonical ubiquitin linkages. These post‐translational codes allow tumor cells to toggle between pro‐invasive and proliferative states, buffer oxidative stress, and remodel cytoskeletal architecture in real‐time. As operationalized in Figure 1 and synthesized in Table 8, context‐specific alterations in mitochondrial structure and function—ranging from fission–fusion dynamics and mitophagy to diverse ubiquitin chain linkages—govern EMT and metastatic progression.

**TABLE 8:** Summary of mitochondrial functional and structural remodeling, mitophagy, and ubiquitination mechanisms influencing EMT and cancer metastasis.

Context	Effect on EMT/Metastasis	Role of ubiquitination
Moderate mtROS levels	Promotes EMT and metastasis via signaling activation	N/A
Excessive mtROS levels	Inhibits metastasis via oxidative damage and ferroptosis	N/A
Increased mitochondrial fission	Promotes EMT and metastasis through cytoskeletal remodeling	Drp1 phosphorylation via ubiquitination‐related pathways
Increased mitochondrial fusion	Supports sustained metabolic demands and metastatic colonization	Regulation of MFN1/MFN2, fusion mediators via ubiquitination
Mitochondrial trafficking to leading edge	Promotes invasion by providing ATP and ROS for motility	N/A
Increased ECM stiffness	Enhances metastatic behavior by modulating mitochondrial dynamics	Indirectly influenced by ubiquitination pathways
Hypoxic conditions	Promotes EMT and invasion via BNIP3/NIX‐dependent mitophagy	N/A
Nutrient deprivation (glucose/amino acid)	Context‐dependent: can enhance invasion or induce stress beyond compensatory capacity	Indirectly regulated via ubiquitin‐mediated pathways (AMPK/mTOR)
Culture dimensionality (3D culture)	Promotes EMT, drug resistance, and metastatic behavior	Enhanced basal mitophagy through ubiquitin‐dependent pathways
Shear stress	Supports extravasation and metastatic survival	Activation of ubiquitin‐dependent mitophagy (Parkin/PINK1)
Stromal cell interaction (CAF/CAM)	Facilitates EMT and metastatic niche formation	Mitochondrial and mtDNA transfer regulated by ubiquitination
Elevated mtDNA content	Supports metastasis via enhanced OXPHOS and bioenergetics	N/A
Reduced mtDNA content and integrity	Context‐dependent: can promote or inhibit metastasis depending on mutation load	N/A
mtUPR activation	Enhances survival, stress resistance, and metastasis	Indirectly activated via stress‐responsive ubiquitination mechanisms
Ubiquitin‐dependent mitophagy (PINK1/Parkin)	Context‐dependent: inhibits early metastatic events, enhances late‐stage progression	Direct ubiquitination of mitochondrial proteins to facilitate autophagy
Receptor‐mediated mitophagy (BNIP3, NIX, FUNDC1)	Context‐dependent: promotes metastatic competence under hypoxic and stress conditions	Direct receptor‐mediated, ubiquitin‐independent LC3 binding
Loss of mitochondrial anchoring (SNPH ubiquitination)	Promotes metastasis by releasing mitochondrial anchoring and facilitating mobility	Direct ubiquitination (K63) of SNPH by CHIP
MICOS complex destabilization	Promotes invasive adaptation by dismantling mitochondrial structure	Ubiquitin‐proteasome mediated degradation of MIC60
Mitochondrial Ca^2^^+^ flux balance (MCU uptake vs NCLX efflux; “Goldilocks zone”)	Biphasic: moderate mitochondrial Ca^2^^+^ supports EMT/invasion (OXPHOS/ATP and redox signaling); Ca^2^^+^ overload or impaired efflux reduces metastatic fitness via mitochondrial stress and cell death	Indirectly influenced by ubiquitination pathways; direct ubiquitin control is better established for ER–mitochondria Ca^2^^+^ transfer regulators (e.g., IP3R3)
Reduced ER‐mitochondria Ca^2+^ transfer (IP3R3 ubiquitination)	Promotes apoptosis‐resistant migratory state enhancing metastasis	Direct ubiquitination (FBXL2) and deubiquitination (BAP1) of IP3R3

The convergence of evidence also underscores the decisive influence of extrinsic cues. Distinct environmental cues—such as hypoxia, amino‐acid starvation, ECM stiffening, fluid shear stress, and soluble factors from stromal cells—encode specific ubiquitin and mitophagy “barcodes” on mitochondrial proteins, which—per magnitude, duration, and tumor identity—either propel or limit dissemination. Such context‐dependency explains the paradoxes in the literature—such as why mitophagy can suppress early metastatic seeding yet support late‐stage colonization, or why mitochondrial fragmentation and fusion can each foster invasion under different mechanical regimes. Prospectively, therapeutic strategies exploiting this stage and site‐specific liability hold considerable promise. Next‐generation PROTACs targeting EMT drivers or lineage‐restricted E3 ligases, small‐molecule activators or blockers of receptor‐mediated mitophagy, and selective DUB inhibitors that rewire mitochondrial ubiquitin codes are poised to complement existing cytotoxic regimens. Key challenges remain—particularly achieving mitochondrial or tumor‐selective delivery and avoiding on‐target toxicity in high‐demand normal tissues. Success will require spatially resolved omics, biomechanical profiling, and organoid‐based assays to customize mitochondrial therapies to the evolving pressures of each tumor. By elucidating the contextual links among ubiquitin signaling, selective mitophagy, and EMT, mitochondrial plasticity can be converted from a driver of metastasis into a druggable weakness.

Outstanding questions and experimental directions. (i) How do defined mechanical niches reprogram E3/DUB activity and mitophagy routing at successive metastatic stages? (ii) Can stage‐restricted targeting of receptor‐mediated versus PINK1/Parkin‐dependent mitophagy suppress dissemination without compromising normal‐tissue MQC? (iii) Which ubiquitin‐linkage “signatures” best predict EMT/MET switching and therapeutic response, and can they be measured in vivo? Answering these questions will help convert mitochondrial plasticity from a driver of metastasis into a druggable weakness.

**BOX 1. Other relevant mitochondrial ion circuits involved in metastasis**

Beyond Ca^2^^+^, mitochondria also regulate other ions—most notably K+ and Cu2+—to fine‐tune bioenergetics, redox balance, and invasive programs of cancer cells. Mitochondrial K^+^ channels—such as ATP‐sensitive (mitoK_ATP_), large‐conductance Ca^2^^+^‐activated (mitoBK_Ca_/K_Ca3.1_), and voltage‐gated K^+^ (mitoKv1.3) isoforms—govern matrix volume, membrane potential, and ROS output, supporting invadopodia formation and motility. Pharmacologic inhibition of mitoKv1.3 selectively kills cancer cells via ROS‐mediated apoptosis and impairs metastasis in melanoma and PDAC models [253]; in parallel, targeting mitoK_Ca3.1_ with mitochondria‐permeant 1‐[(2‐chlorophenyl) diphenylmethyl]‐1H‐pyrazole derivatives (designated as TRAM‐34) depolarizes the mitochondrial inner membrane, elevates mtROS, and suppresses tumor migration and metastasis in preclinical models [254].

Moreover, mitochondria regulate intracellular copper distribution via the antioxidant 1 copper chaperone (ATOX1), the cytochrome c oxidase copper chaperone (COX17), as well as copper‐transporting ATPases ATPase copper‐transporting alpha and beta (ATP7A and ATP7B), in this way controlling copper‐dependent enzymes that remodel the ECM and drive angiogenesis. Disrupted mitochondrial copper handling can enhance lysyl oxidase (LOX) family activity, promoting collagen crosslinking, matrix stiffening, and increased invasiveness in estrogen receptor‐negative breast cancer [253, 255]. When forced beyond a threshold, copper ions can provoke cuproptosis—a mitochondria‐linked, copper‐dependent form of regulated cell death propelled by aggregation of lipoylated TCA‐cycle proteins and proteotoxic stress. Therapeutically, cupric ionophores and copper‐chelation strategies can leverage this liability to preferentially eliminate highly invasive or therapy‐evasive cancer cells, suggesting an emerging anti‐metastatic paradigm [256, 257].

**BOX 2. Could histone DUBs regulate mtUPR?**

Although DUBs classically erase ubiquitin marks on cytosolic and organellar substrates, mtUPR gene‐expression induction is also chromatin‐gated; thus, nuclear histone‐directed DUBs such as BAP1 and Myb‐like, SWIRM, and MPN domains 1 (MYSM1) could in principle redefine the mtUPR transcriptional “set point” through chromatin remodeling [258]. In *Caenorhabditis elegans*, mtUPR engagement is orchestrated through a sequential histone‐marking cascade—H3K9 methylation by methyltransferase‐2 (MET‐2), H3K27 demethylation by Jumonji domain‐containing proteins 1.2 and 3.1 (JMJD‐1.2 and JMJD‐3.1), and histone deacetylation by histone deacetylase 1 (HDA‐1)—which primes stress‐responsive loci for recruitment of the defective proventriculus‐1 (DVE‐1)/ubiquitin‐like protein 5 (UBL‐5) transcriptional complex. In mammalian systems, one concrete candidate for parallel chromatin gating is histone H2A Lys119 monoubiquitination (H2AK119ub1; H2Aub), added by polycomb repressive complex 1 (PRC1) and reversed by H2A‐directed DUBs such as USP16 and USP21; recent structural and regulatory work has begun to characterize how USP16 engages H2AK119ub1 nucleosomes and how its activity can be modulated, providing a mechanistic foothold for testing mtUPR‐linked chromatin tuning [259]. Whether this chromatin‐ layer ubiquitin editing meaningful tunes mtUPR output in human cancer remains unresolved, but it offers a testable route whereby nuclear stress‐responsive transcription could be coupled to motility programs, extending the organelle‐proximal ubiquitin logic outlined in Section 4.2.

## Author Contributions

B.H.M. conceptualized the work, and Y.J.W. and T.Y.T provided direction and guidance throughout the preparation of this manuscript. B.H.M. and B.K.S. collated the literature, wrote the manuscript and created the figures. All authors have read and approved the final manuscript.

## Conflicts of Interest

The authors declare no competing interests.

## Supporting information




**Supporting File**: advs74078‐sup‐0001‐SuppMat.docx.

## Data Availability

The authors have nothing to report.
