# Supplementary material for: Mitophagy and Ubiquitination Coordinate Context‐Specific Mitochondrial Quality Control and EMT/MET Plasticity to Drive Cancer Cell Invasion
Source: Adv Sci (Weinh). 2026 Feb 17;13(16):e19792. doi: 10.1002/advs.202519792 (PMC13042459; doi:10.1002/advs.202519792)
Supplement: Supplementary file 1 — Supporting File: advs74078‐sup‐0001‐SuppMat.docx. [file ADVS-13-e19792-s001.docx]

| **Table S1. Emerging pharmacologic modulators of the ubiquitin–mitophagy axis: targets, regulatory mechanisms, and evidence stage** | | | | | | |
| --- | --- | --- | --- | --- | --- | --- |
| Category | Agent / Modality | Primary target | Regulatory mechanism (succinct) | Key mitophagy/UPS readouts reported | Notes for cancer/EMT-mets framing | Refs |
| Mitophagy inducer (natural postbiotic) | Urolithin A (UA) | Indirect / pleiotropic (mitochondrial QC program activation) | Promotes mitophagy and mitochondrial remodeling; reported to improve mitochondrial function and stress resilience in multiple models | Mitophagy markers/flux ↑; improved mitochondrial respiration/fitness readouts in vivo | Cancer/EMT/metastasis-specific evidence varies by context; cite conservatively (do not overclaim anti-metastatic effects unless directly shown) | (262-264) |
| DUB inhibitor (mitochondrial outer membrane) | FT3967385 (FT385) – USP30 inhibitor | USP30 (deubiquitinase) | Inhibits USP30 to lower the threshold for PINK1–Parkin amplification; increases ubiquitination of mitochondrial substrates (e.g., TOM complex components) | TOMM20 ubiquitination ↑; phospho‑S65‑Ub accumulation ↑; mitophagy signatures after depolarization ↑ | Translational node: removes a 'brake' on ubiquitin‑mitophagy; note current evidence base is mainly neuro/mitophagy models | (265) |
| DUB inhibitor (mitochondrial outer membrane) | CMPD-39 (Compound 39) – USP30 inhibitor | USP30 (deubiquitinase) | Selective USP30 inhibition enhances mitophagy and pexophagy; phenocopies USP30 loss in multiple assays | Mitolysosome number/size ↑; TOMM20 & SYNJ2BP ubiquitination ↑; mitophagy ↑; rescue of mitophagy in PRKN-mutant neurons reported | If you discuss EMT/metastasis: frame as 'drugging the ubiquitin‑mitophagy brake' rather than claiming direct anti‑metastatic efficacy | (265) |
| PINK1 activator (neo-substrate strategy) | Kinetin → Kinetin triphosphate (KTP) (neo-substrate) | PINK1 kinase | KTP acts as a neo-substrate to enhance PINK1 catalytic activity; kinetin is metabolic precursor that boosts PINK1 activity in cells | Parkin recruitment to damaged mitochondria ↑; PINK1-dependent signaling outputs ↑ (per paper assays) | Useful to illustrate druggable PINK1 activation logic; avoid implying clinical readiness | (266) |
| PINK1 activator / stabilizer (small molecule) | MTK458 (Mitokinin/AbbVie; PINK1 activator) | PINK1 kinase | Binds to PINK1 and stabilizes an active complex/heterocomplex; increases mitophagy rates | Mitophagy ↑; pS65‑Ub modulation; reduced α-syn pathology readouts in PD models (preclinical) | For cancer context: present as an example of direct PINK1 pharmacological activation; clearly label evidence domain (neuro/PD) | (267) |
| PINK1 stabilization via protease inhibition | PARL ketoamide inhibitors (e.g., 'compound 5' in paper) | PARL (mitochondrial rhomboid protease) → stabilizes PINK1 by blocking cleavage | Acute PARL inhibition stabilizes PINK1 intermediates and robustly activates PINK1/Parkin pathway | PINK1 stabilization ↑; Parkin activation ↑; pathway activation without major secondary mitochondrial property changes (per authors) | For EMT/metastasis discussion: emphasize 'PINK1 stabilization node' and potential for context-dependent effects | (9) |
| Mitophagy inducer via PINK1/Parkin activation | BL-918 (reported mitophagy inducer; also ULK1 activator literature exists) | PINK1/Parkin pathway activation (mechanism involves mPT-associated depolarization in paper) | Triggers PINK1 accumulation and Parkin translocation; activation involves mitochondrial depolarization/mPT pore per study | PINK1 accumulation ↑; Parkin translocation ↑; ubiquitin phosphorylation ↑; mitophagy markers ↑ | For cancer context: classify as pathway activator; avoid using as 'targeted' unless you also discuss its reported ULK1 activity | (268) |
